# HER2 and p95HER2 differentially regulate miRNA expression in MCF-7 breast cancer cells and downregulate MYB proteins through miR-221/222 and miR-503

**DOI:** 10.1038/s41598-019-39733-x

**Published:** 2019-03-04

**Authors:** Andrej Gorbatenko, Rolf Søkilde, Ester E. Sorensen, Inga Newie, Helena Persson, Beatriz Morancho, Joaquin Arribas, Thomas Litman, Carlos Rovira, Stine Falsig Pedersen

**Affiliations:** 1 Department of Pathology, Icahn School of Medicine at Mount Sinai, New York, New York, 10029 USA; 2 Section for Cell Biology and Physiology, Department of Biology, Faculty of Science, University of Copenhagen, Universitetsparken 13, DK-2100 Copenhagen, Denmark; 3 BioCare, Strategic Cancer Research Program, Lund, Sweden; 4 Department of Clinical Sciences Lund, Oncology and Pathology, Faculty of Medicine, Lund University, Lund, Sweden; 5 Preclinical Research Program, Vall d’Hebron Institute of Oncology and CIBERONC, 08035 Barcelona, Spain; 6 Department of Biochemistry and Molecular Biology, Universitat Autonoma de Barcelona, Campus de la UAB, JA Bellaterra, Spain; 7 Institució Catalana de Recerca i Estudis Avançats, JA Barcelona, Spain; 8 Department of International Health, Immunology and Microbiology, University of Copenhagen, Blegdamsvej 3B, DK-2200 Copenhagen, Denmark

## Abstract

The HER2 oncogene and its truncated form p95HER2 play central roles in breast cancer. Here, we show that although HER2 and p95HER2 generally elicit qualitatively similar changes in miRNA profile in MCF-7 breast cancer cells, a subset of changes are distinct and p95HER2 shifts the miRNA profile towards the basal breast cancer subtype. High-throughput miRNA profiling was carried out 15, 36 and 60 h after HER2 or p95HER2 expression and central hits validated by RT-qPCR. miRNAs strongly regulated by p95HER2 yet not by HER2, included miR-221, miR-222, miR-503, miR-29a, miR-149, miR-196 and miR-361. Estrogen receptor-α (ESR1) expression was essentially ablated by p95HER2 expression, in a manner recapitulated by miR-221/-222 mimics. c-Myb family transcription factors MYB and MYBL1, but not MYBL2, were downregulated by p95HER2 and by miR-503 or miR-221/-222 mimics. MYBL1 3′UTR inhibition by miR-221/222 was lost by deletion of a single putative miR-221/222 binding sites. p95HER2 expression, or knockdown of either MYB protein, elicited upregulation of tissue inhibitor of matrix metalloprotease-2 (TIMP2). miR-221/222 and -503 mimics increased, and TIMP2 knockdown decreased, cell migration and invasion. A similar pathway was operational in T47D- and SKBr-3 cells. This work reveals important differences between HER2- and p95HER2- mediated miRNA changes in breast cancer cells, provides novel mechanistic insight into regulation of MYB family transcription factors by p95HER2, and points to a role for a miR-221/222– MYB family–TIMP2 axis in regulation of motility in breast cancer cells.

## Introduction

The receptor tyrosine kinase HER2 (ErbB2) is overexpressed or amplified in 20–30% of breast cancer patients, correlating with cancer aggressiveness and reduced patient survival^1^. About 30% of patients also express a constitutively active form of HER2, known as p95HER2, lacking the extracellular domain and associated with increased aggressiveness, Herceptin (trastuzumab) resistance in monotherapy, and poor prognosis^2–4^. MicroRNAs (miRNAs) are widely implicated in cancer development, acting either as promoters (oncomiRs) or suppressors of disease^5,6^. Altered miRNA levels are increasingly investigated for diagnostic use in various cancers including breast cancer^5,7^, and several miRNA-targeting drugs, such as the miR-122 inhibitor Miravirsen^8^ and the miR-34 mimic MRX34 (Phase I study NCT01829971) have entered clinical trials. The roles of miRNA dysregulation in breast cancer have been widely studied, and characteristic miRNA signatures have been explored for different breast cancer subtypes including HER2 overexpressing cancers^9,10^. However, the possibility that HER2 and p95HER2 may elicit different changes in miRNA expression has, to our knowledge, never been addressed. The two related miRNAs miR-221 and miR-222, which have been implicated in numerous aspects of breast cancer pathology^11–13^ were reported to be upregulated in HER2-positive primary human breast cancer tissue^11^, and miR-221-HER2 co-expression was shown to be a negative prognostic marker for distant metastasis-free survival^14^. miR-221 and -222 expression negatively correlates with Estrogen Receptor-α (ESR1) status due to downregulation of ESR1 by these miRNAs^12^.

The *v-myb avian myeloblastosis* viral oncogene homolog (MYB) family of transcription factors comprises MYB (c-MYB), MYB-like-1 (MYBL1, A-MYB) and MYBL2 (B-MYB)^15–17^. While they share similar DNA binding domains and bind to the same DNA sequences, the three family members activate partially distinct sets of genes^17,18^ and their knock-out mouse models elicit distinct phenotypes (see^15^). While only the viral, truncated form of c-MYB, v-MYB, appears to be a *bona fide* oncogene, the normal cellular counterparts are also implicated in cancer development, although their roles remain incompletely understood. c-MYB is overexpressed or mutated in a variety of cancers, including breast cancer, where its expression generally correlates with that of ESR1^19^ because ESR1 signaling positively regulates MYB expression^20^. This is functionally highly significant, as MYB silencing blocks estrogen-dependent breast cancer cell proliferation^20^. In addition, MYB is amplified in 30% of BRCA1 mutant hereditary breast cancers^21^. MYB is extensively regulated at the transcriptional, posttranscriptional and posttranslational levels^15^. The 3′UTR of MYB has several putative miRNA binding sites, and has been shown to be subject to miRNA mediated regulation^15^, specifically as a target of miR-503^22^. MYBL1 is deregulated in several leukemias^23^, and MYBL1 translocations are associated with adenoid cystic carcinomas^24^ and gliomas^25^. MYBL1 activity is cell cycle dependent and regulated by cyclins A and E^26^. Notably, miR-221 was suggested to negatively control expression of MYBL1 in liver cancer^27^. MYBL2 expression is upregulated in several cancers, including breast cancer^28,29^, reportedly with the highest expression in basal-like breast cancer, and lowest in normal-like and luminal A type breast cancer^28^. MYBL2 is upregulated during cell cycle progression, peaking in S-phase, and is an essential regulator of G2/M progression and cell proliferation^30^. MYBL2 has been assigned important roles in regulating entry into senescence^31^ and has been shown to rescue oncogene-induced senescence in cells overexpressing activated *ras*^32^. This is particularly interesting because p95HER2 overexpression has been shown to be a potent inducer of senescence in breast cancer cells, albeit in a manner that may be associated with overall increased aggressiveness due to senescence-associated secretion of pro-tumorigenic factors^33^. Similar to MYB, both MYBL1 and MYBL2 are upregulated by ESR1 signaling in breast cancer cells^34^.

The aim of this work was to gain insight into the mechanisms underlying the functional differences between HER2 and p95HER2 in breast cancer. We employed next generation sequencing to study the miRNA expression profiles induced by these HER2 variants and we specifically interrogated whether p95HER2-induced cellular traits associated with breast cancer aggressiveness involve p95HER2 mediated regulation of miR-221/222 and miR-503 and in turn MYB family transcription factors. We demonstrate that p95HER2 impacts a subset of miRNAs much more strongly than does HER2 and causes a shift toward the miRNA profile associated with basal subtype breast cancers. Highly p95HER2-upregulated miRNAs include miR-221-/222, resulting in essential ablation of ESR1 expression in p95HER2-expressing cells, and miR-503, which together with miR-221/222 elicit downregulation of MYB and MYBL1. This in turn leads to upregulation of of the putative MYB family target gene, tissue inhibitor of matrix metalloproteases-2 (TIMP2). Finally, using miR mimic overexpression and TIMP2 siRNA knockdown, we show that a miR-221/222, miR-503 → MYB, MYBL1 → TIMP2 axis stimulates migration and invasion of MCF-7 breast cancer cells. Similar results were obtained in T47D- and SKBr-3 breast cancer cells. Our findings identify a novel role for p95HER2-specific miRNA changes in distinguishing p95HER2 positive cancers from those overexpressing only HER2, and uncover a signaling axis from miR-221/222 and -503 over MYB proteins to TIMP2 important for controlling cell motility in breast cancer.

## Results

### HER2 and p95HER2 elicit overlapping, but distinct changes in miRNA expression in MCF-7 cells

**Figure 1:**
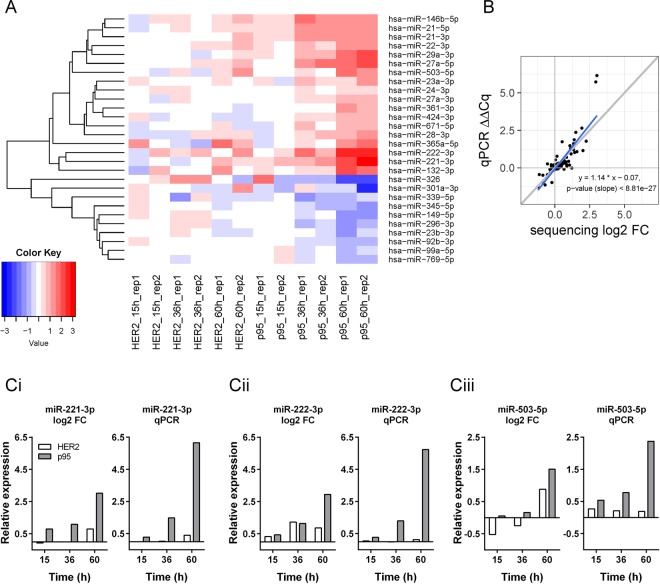
Overview of the miRNA sequencing data and validation of selected miRNAs. (**A**) The heat-map shows the log2 fold change ratios between minus (induced) and plus doxycycline conditions. Expression values are log counts per millions +2 pseudo-counts after normalization with TMM (weighted trimmed mean of M-values) in the edgeR package. Euclidian distance and average linkage method were used to generate the clusters of miRNAs using the gplots package in R. There are 28 miRNAs in the clustering of which 10 are downregulated, while 18 are upregulated. There is generally good agreement between the replicate samples in the experiments. (**B**) Correlation between sequencing and qPCR results. Comparison is done with log2 fold change (FC) and ΔΔCq for all the relevant comparisons between +/− dox. Grey diagonal line indicates a slope of 1, while the blue line is the fit to the data. The slope of the fit is 1.14 and highly significant, indicating a very strong correlation between the platforms. The two points on the top of the blot are miR-221 and miR-222, which deviate from the ideal slope due to a greater increase seen in qPCR than in sequencing. (**C**) Comparison of sequencing and qPCR data for miR-221-3p (Ci), miR-22-3p (Cii) and miR-503-5p (Ciii). Values are the same as those used in (**B**).

In order to investigate the global effects of HER2 and p95HER2 on miRNA expression, we employed MCF-7 human breast cancer cells inducibly expressing the full length HER2 and p95HER2 coding regions, respectively^3^. Samples were collected 15, 36 and 60 h after doxycycline removal to induce expression, or in the corresponding uninduced cell lines as controls (Suppl. Fig. 1). All significantly altered miRNAs are listed in Suppl. Table 2 with log2 fold changes relative to control. Of the 28 differentially expressed miRNAs, 10 were down-regulated in the p95HER2 expressing cells at the 60 h time point, while 18 were up-regulated. A heat-map of the significantly altered miRNAs is shown in Fig. 1A. qPCR validation was performed for 16 of the significantly regulated miRNAs, and showed excellent correlation with the sequencing results (Fig. 1B). As expected, HER2 and p95HER2 induced qualitatively similar changes in the global miRNA profile in the MCF-7 cells, yet with several distinct differences. miRNAs regulated by both variants were generally most strongly up-/downregulated by p95HER2 and some were essentially unaffected by HER2. 12 of the 16 miRNAs were upregulated by p95HER2 (miR-21-3p, miR-22-3p, miR-27a-3p, miR-27a-5p, miR-28-3p, miR-29a-3p, miR-132-3p, miR-146b-5p, miR-503-5p, miR-221-3p, miR-222-3p, miR-361-3p) while 4 were downregulated (miR92b-3p, miR-149-5p, miR-296-3p, miR-769-5p) (Fig. 1C; Suppl. Fig. 2). It is noteworthy that the magnitude of the effects of HER2 and p95HER2 on most of these miRNAs differed robustly, with generally several fold greater effect of p95HER2. This was especially marked at time 60 h, and for further analyses, we focused our attention on this time point.

### Correlation between p95HER2 expression, miRNA profile, and breast cancer subtype

**Figure 2:**
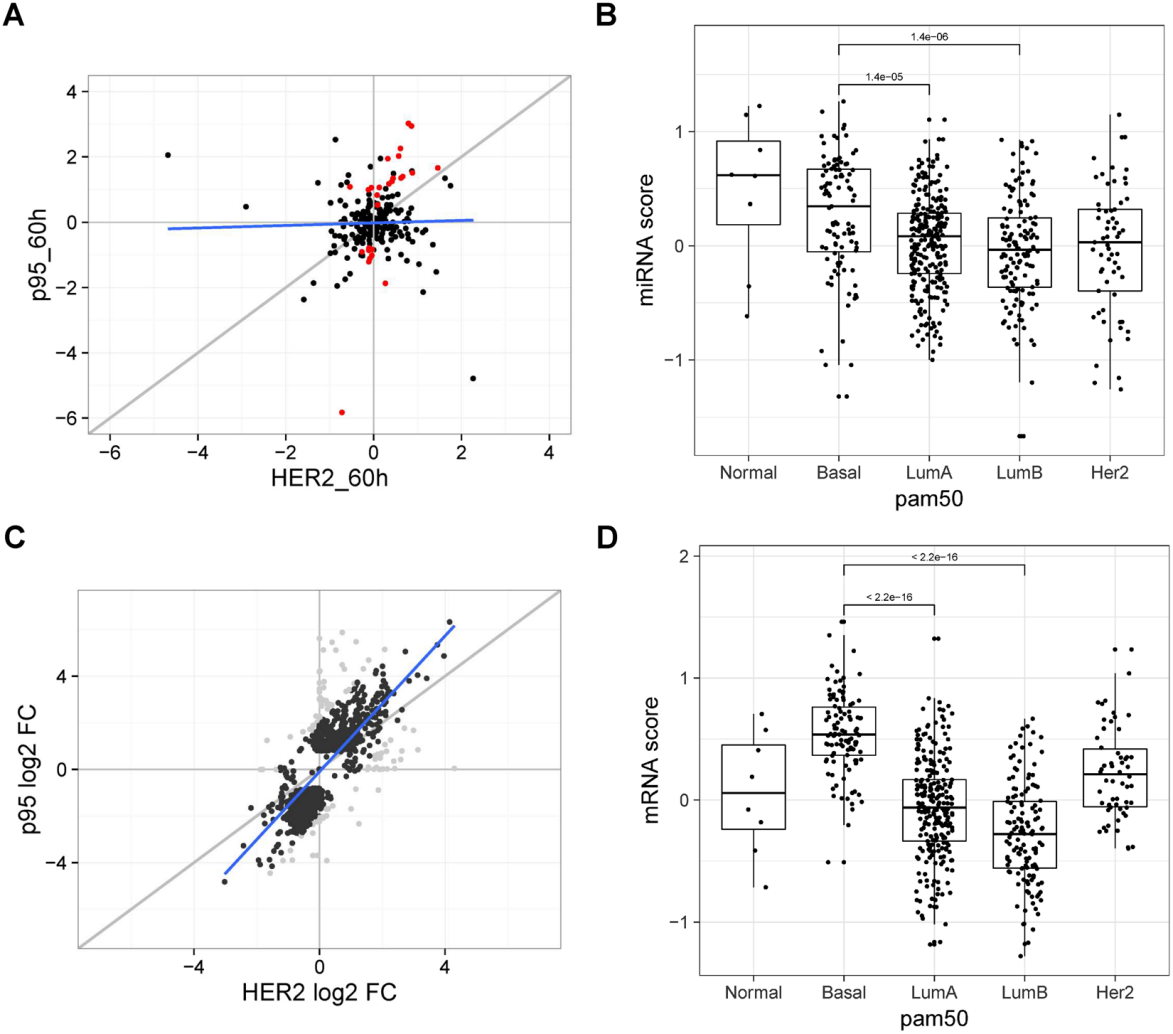
miRNA and mRNA scores differ between basal and luminal breast cancers. (**A,B**) are plots related to miRNAs, while (**C,D**) are related to mRNAs. Plots in A and C are based on data from the present study and from *GSE68256*, respectively, data in B are from TCGA and data in C from^35^. We identified 18 up-regulated and 10 down-regulated miRNAs, which are significantly deregulated in p95 samples and not in HER2 samples (red points). While there is a tight relationship between mRNAs upregulated in p95HER2 and HER2 (slope = 1.46, p-value = 5.06e-142) (panel C), surprisingly we did not find the same relationship for miRNAs (slope = 0.0442, p-value = 0.375) (panel A). We used the significant miRNAs from the present study to calculate a miRNA score: *miRNA Score* = *mean(up-regulated miRNAs)* − *mean(down-regulated miRNAs)*. This score was calculated for all samples in the TCGA cohort and we found a significantly (Wilcoxon) greater score for samples from basal compared to luminal tumor types (PAM50). There are 50 downregulated and 53 upregulated mRNAs in the mRNA score calculated from *GSE68256* as: *mRNA Score* = *mean(filtered up-regulated mRNAs) − mean(filtered down-regulated mRNAs)*. For both miRNA (**B**) and mRNA (**D**), scores were higher in basal than in luminal samples indicating that both mRNAs and miRNAs deregulated in the MCF-7 cells (luminal) are involved in the basal signature for breast cancer.

We identified 18 up-regulated and 10 down-regulated miRNAs, which were significantly deregulated in p95HER2 samples but not in HER2 samples (Suppl. Table 2). To assess how the effects of HER2 and p95HER2 upregulation on miRNA levels in MCF7 cells compare to their effects on mRNA levels, we analyzed the correlations between miRNA and mRNA levels in HER2 and p95HER2 expressing cells, using miRNA data from the present study (Fig. 2A) and mRNA data from *GSE68256*^3^, which was obtained using the same cells and experimental setup (Fig. 2C). Notably, the tight relationship between mRNAs upregulated by p95HER2 and HER2 is not seen for miRNAs. Next, we therefore used the miRNAs significantly altered by p95HER2 to calculate a microRNA score [mean(up-regulated miRNAs) − mean(down-regulated miRNAs] for all samples in the TCGA cohort^35^, revealing a significantly (Wilcoxon) greater score for samples from basal compared to luminal tumor types (PAM50). Similarly, a mRNA score was calculated as [mean(filtered up-regulated mRNAs) − mean(filtered down-regulated mRNAs)] (see Materials and Methods) and applied to the TCGA cohort data. Similar to the miRNA scores, the mRNA scores were higher in the basal samples.

Collectively, these data indicate that mRNAs and miRNAs deregulated in the cell line (MCF7, luminal) are involved in the basal signature for breast cancer.

### p95HER2 downregulates ESR1 protein expression via miR-221/222

**Figure 3:**
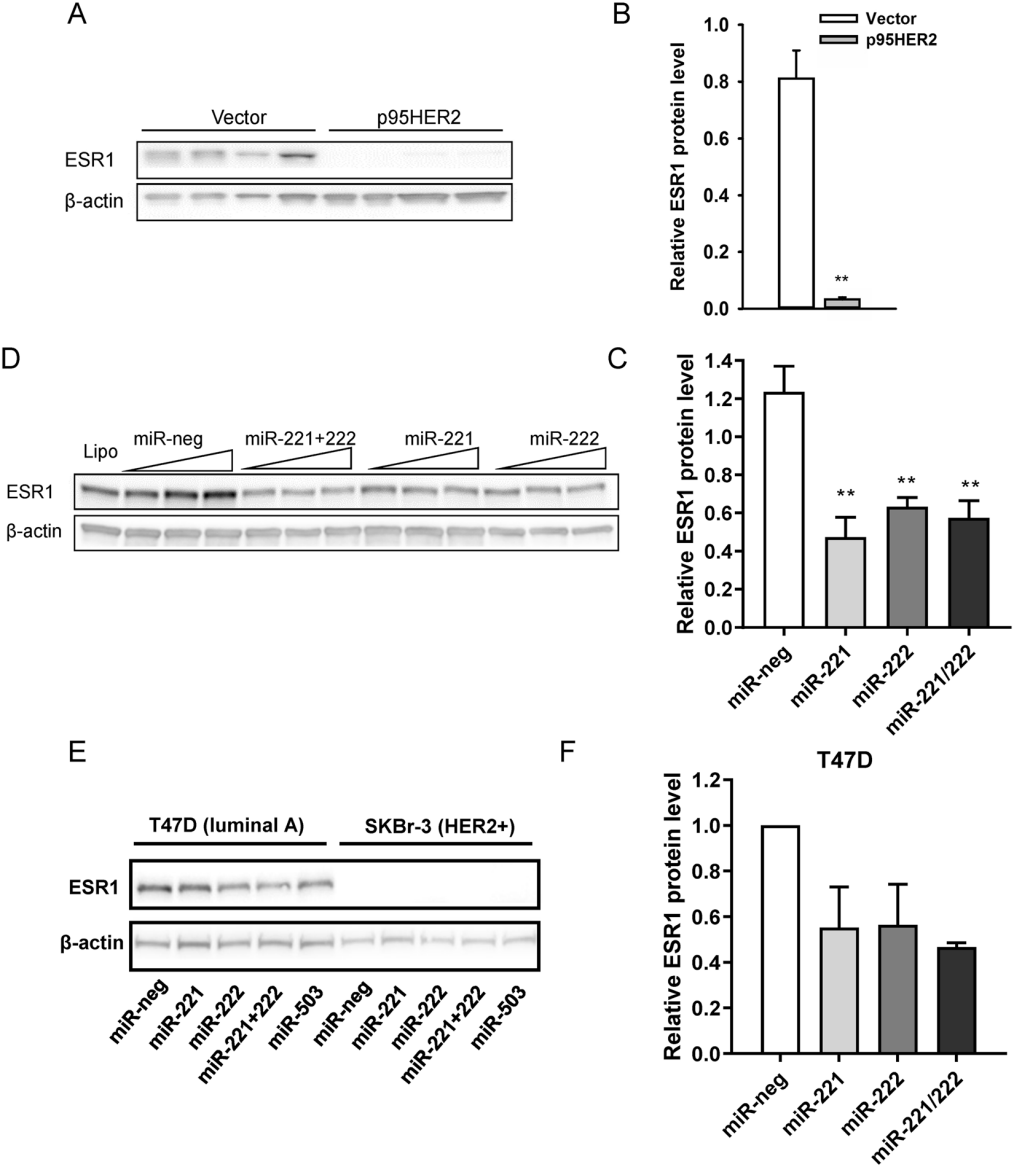
Inhibition of ESR1 protein expression by p95HER2 and miR-221 and -222. (**A,B**) Vector- and p95HER2-expressing MCF-7 cells were lysed at time 48 h after induction, and subjected to Western blot analysis of ESR1 expression. β-actin is shown as a loading control. The graph shows summarized data as mean values with S.E.M. error bars of 4 independent, parallel experiments for each cell line, normalized to the β-actin loading control. **Significantly different from vector control, p < 0.01. (**C,D**) Immediately after induction, cells were transfected with miR-221, miR-222, or negative control miRNA mimics (20, 40 and 80 nM final concentrations respectively). 48 h later, cells were lysed and subjected to Western blot analysis of ESR1 expression. β-actin is shown as a loading control. The graph shows mean values from 40 nM transfections, with S.E.M. error bars of 5 independent, parallel experiments, normalized to the β-actin loading control. **Significantly different from negative control mimic, p < 0.01. (**E,F**) The representative blot shows T47D and SKBr-3 cells subjected to Western blot analysis of ESR1 expression. p150^Glued^ is shown as a loading control. The graph shows mean values with S.E.M. error bars of 2 independent experiments for T47D cells, normalized to the β-actin loading control.

Next, to identify mechanisms through which p95HER2-regulated miRNAs might exert functional effects, we searched for potential targets in previously published microarray data of changes in gene expression induced by HER2 or p95HER2 at 15 and 60 h after expression induction, i.e. directly comparable to our small RNA sequencing data^3^. Of note, ESR1, which has been shown to be negatively regulated by miR-221/222^12^, was downregulated by p95HER2 in this dataset. Remarkably, ESR1 protein expression was essentially abolished upon p95HER2 overexpression (Fig. 3A,B). This effect was partially reproduced by transfection of vector expressing MCF-7 cells with miR-221 and/or -222 mimics (Fig. 3C,D), showing that ESR1 downregulation in p95HER2 MCF-7 cells at least in part reflects upregulation of miR-221 and -222. A similar downregulation of ESR1 protein level by miR-221 and -222 was observed in T47D luminal A type breast cancer cells, whereas ESR1 expression is undetectable in the HER2+ subtype SKBr-3 cells (Fig. 3E,F). Taken together, these data point to important interactions between HER2/p95HER2 and ESR1, which can influence downstream signaling effects and hence disease progression. A recent report showed that ERK1/2 MAPK hyperactivation (denoted hMAPK), which is associated with ESR1 downregulation and poor outcome in breast cancer, can be linked to a characteristic miRNA signature^36^. We therefore compared the miRNA signature obtained in our study to the hMAPK signature reported by Miller *et al*.^36^ (Suppl. Fig. 3). As seen, there is a striking overlap between the p95HER2 induced miRNA changes and the hMAPK signature, in agreement with the profound ESR1 downregulation and strong upregulation of ERK1/2 signaling in p95HER2 overexpressing MCF-7 cells^37^.

### p95HER2 downregulates MYBL1 protein expression via miR-503 and miR-221/222

**Figure 4:**
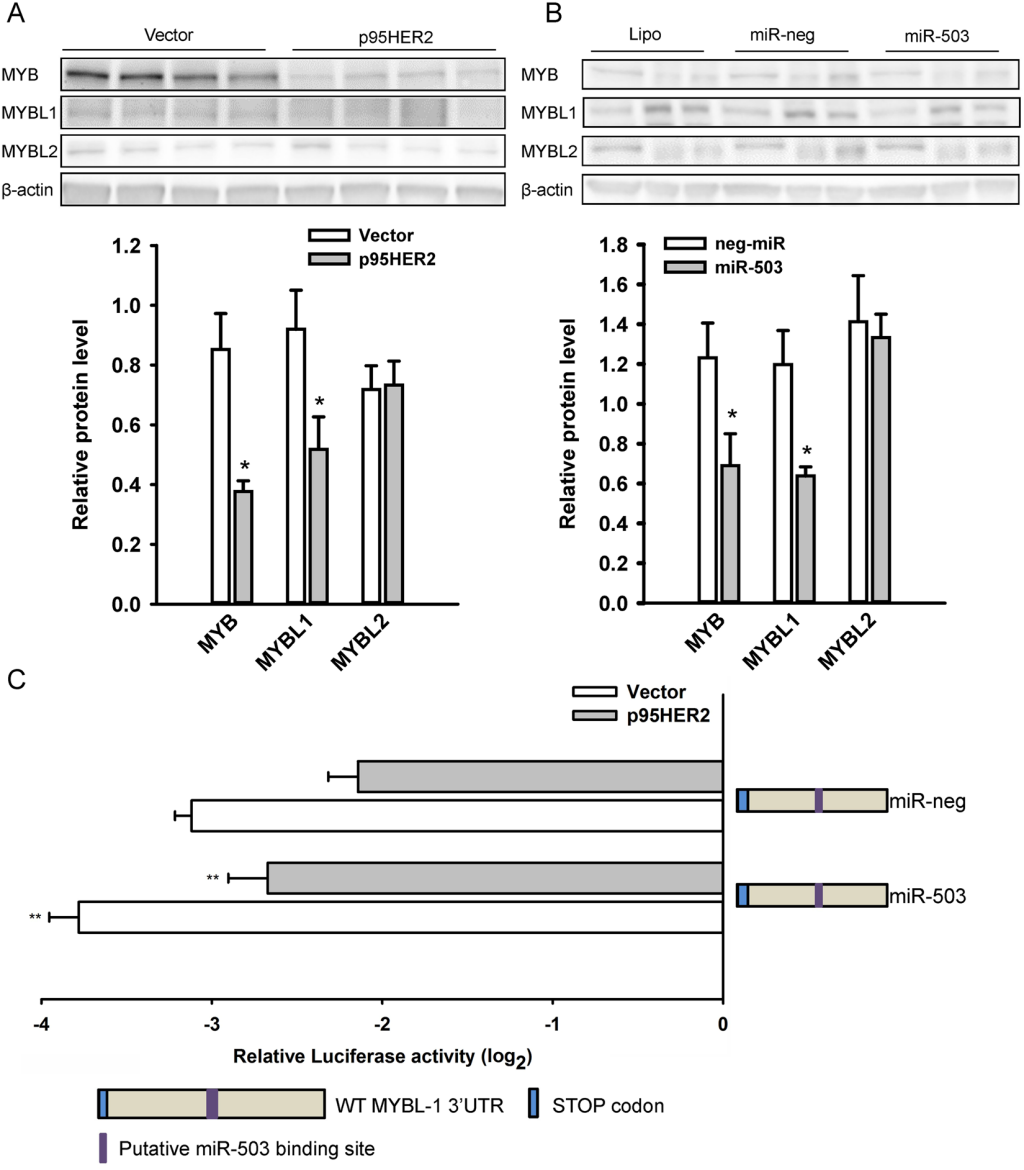
Inhibition of MYB and MYBL1 protein expression by p95HER2 and miR-503. (**A**) MYB family protein expression in vector- and p95HER2-overexpressing cells. Cells were lysed 48 h after induction and subjected to Western blotting. β-actin is shown as loading control. The graph shows mean values with S.E.M. error bars of 5 independent, parallel experiments, normalized to the β-actin loading control. *Significantly different from the corresponding value in vector cells, p < 0.05. (**B**) Effect of miR-503 mimics on MYB family protein expression in vector cells. Immediately after induction, cells were transfected with miR-503 or negative control miRNA mimics. 48 h later, cells were lysed and subjected to Western blot analysis of MYB, MYBL1 and MYBL2 expression. β-actin is shown as a loading control. The graph shows mean values with S.E.M. error bars of 3 independent, parallel experiments, normalized to the β-actin loading control. *Significantly different from the corresponding value in vector cells, p < 0.05. (**C**) Effect of miR-503 mimics on MYBL1 3′UTR regulation of upstream renilla luciferase activity. The psiCHECK2 plasmid containing the wild-type 3′UTR of MYBL1 was cotransfected into vector and p95HER2 cells with 40 nM of miR-503 or negative control miRNA mimics. 48 h after transfection, cells were lysed and luciferase activity measured as described in Materials and Methods. Data was normalized to firefly luciferase activity and are shown as the log2 transformed change relative to empty psiCHECK2 vector. The graph shows mean values with S.E.M. error bars of 11–12 independent, parallel experiments. * and **Significantly different from corresponding negative control miRNA values, p < 0.05 and 0.01, respectively.

Another gene markedly downregulated by p95HER2 in the GSE68256 dataset was the transcription factor MYBL1. Furthermore, an Integrated System for Motif Activity Response Analysis (ISMARA) analysis^38^ of our miRNA sequencing dataset predicted the activity of the closely related transcription factor MYB to be markedly reduced after p95HER2 expression (data not shown). Thus, we focused our analyses on the MYB family, consisting of MYB, MYBL1, and MYBL2. We first asked whether the protein levels of these transcription factors were altered by p95HER2 expression (Fig. 4A). As predicted by the GSE68256 dataset and ISMARA analysis, MYB and MYBL1 protein levels were significantly downregulated by p95HER2. In contrast, the protein level of MYBL2 was unaffected. Analysis of the MYB and MYBL1 3′UTRs revealed putative binding sites for miR-503 in both, whereas MYBL2 lacked such sites. Given the robust upregulation of miR-503 by p95HER2 (Fig. 1C), we transfected vector cells with a miR-503 mimic. Importantly, this mimicked the effect of p95HER2 on expression of MYB family members, i.e. resulted in a reduction of MYB and MYBL1 expression, while MYBL2 was unaffected (Fig. 4B). A similar effect of miR-503 mimics on MYBL1 was seen in T47D and SKBr-3 cells (Suppl. Figs 4 and 5).

Next, to determine the role of the putative miR-503 binding site in MYBL1, the 3′UTR activity of MYBL1 was determined using a psiCHECK2 luciferase reporter assay (Fig. 4C). The presence of p95HER2 modestly *in*creased luciferase activity compared to that in vector cells, in apparent contrast to the effect on MYBL1 protein expression (compare with panel A). A similar effect was noted recently in another study using the psiCHECK2 system, and we speculate that it reflects a p95HER2-mediated enhancer effect on Renilla luciferase^39^. Importantly however, in both vector- and p95HER2-expressing cells, introduction of a miR-503 mimic exacerbated the inhibitory effect of the MYBL1 3′UTR on luciferase activity compared to a negative control miRNA mimic.

**Figure 5:**
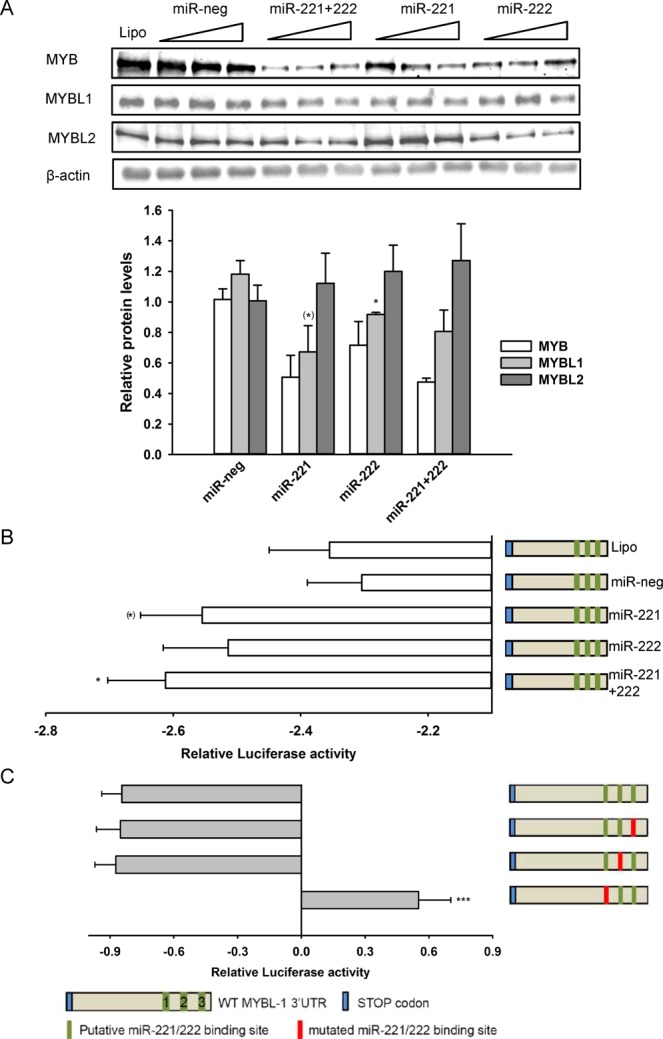
miR-221/222 downregulates MYBL1 through direct interaction with the MYBL1 3′UTR. (**A**) Vector MCF-7 cells were transfected with 10, 20 or 40 nM (indicated by the triangles) miR-221 or miR-222, both mimics combined, or negative control miRNA as shown, lysed, and subjected to Western blotting for MYB family protein expression as indicated. The graph shows the means of the 40 nM miRNA data, with S.E.M. error bars, normalized to the β-actin loading control. Data is from 3 biological replicates. (**B**) Effect of miR-221 and miR-222 mimics on MYBL1 3′UTR activity. The wild-type 3′UTR of MYBL1 was cloned into the psiCHECK2 plasmid just downstream renilla luciferase, and co-transfected with 40 nM of the indicated miRNA mimics into vector control cells. 48 h after transfection, cells were lysed and renilla and firefly luciferase activity measured as described in Materials and Methods. Renilla luciferase activity was normalized to that of firefly luciferase and is shown as the log2 transformed change relative to empty psiCHECK2 vector. The graph shows mean values with S.E.M. error bars of 18 independent, parallel experiments. (**C**) Effect of deletion of putative miR-221/222 binding sites on MYBL1 3′UTR activity in p95HER2 cells. The psiCHECK2 vector containing wild-type MYBL1 or this construct with disruptive mutations introduced in each of the three putative miR-221/222 binding sites were transfected into p95HER2 cells. Renilla luciferase activity was normalized to Firefly luciferase activity and is shown as the log2 transformed change relative to empty psiCHECK2 vector. ***P < 0.001, significantly different from wild type 3′UTR. Data is from 6–18 biological replicates.

Similar to ESR1, the 3′ UTRs of MYBL1 and MYB, but not of MYBL2, also exhibited putative binding sites for miR-221/222. The effect of miR-221/222 on MYBL1 protein expression was determined in a similar way. Transfection of vector cells with mimics of miR-221, miR-222 or both, resulted in modestly reduced MYB and MYBL1 protein levels, while expression of MYBL2 was unaffected (Fig. 5A). In accordance with this, all three miRNA conditions tended to augment the inhibitory effect of the MYBL1 3′ UTR on luciferase activity (Fig. 5B). To assess the roles of the putative miR-221/222 binding sites in the regulation of MYBL1 by miR-221/222, each of the 3 binding sites was mutated and their effect on the MYBL1 3′UTR determined (Fig. 5C). Notably, mutation of the first (closest to the ORF) miR-221/222 binding site completely abolished 3′UTR mediated downregulation of luciferase activity in p95HER2 cells. In fact, removal of this binding site switched the effect of the 3′UTR from its normal negative regulatory effect to an upregulation of the relative Renilla activity, whereas removal of the two other sites had no effect. The inhibitory effect of miR-221 and -222 on MYB and MYBL1 expression was not restricted to MCF-7 cells: A similar response was observed in T47D cells as models of Luminal A breast cancer cells (Suppl. Fig. 4). Further, MYBL1 expression was profoundly inhibited by these miRs in SKBr-3 cells (modeling HER2+ breast cancer cells) (Suppl. Fig. 5), which do not express MYB to detectable levels (not shown).

Finally, we analyzed TCGA data for mRNA expression of MYB, MYBL1 and MYBL2 in normal, basal, luminal A, luminal B, and HER2 positive patient samples (Suppl. Fig. 6). Interestingly, this analysis revealed that the MYB mRNA level was strongly reduced in the basal cancer subtype, and those of MYBL1 and MYBL2 increased, compared to the levels in normal tissue, partially in line with our finding that p95HER2 expression shifts the miRNA profile towards this subtype (Fig. 2). Moreover, the mRNA level of MYBL2 was elevated in HER2 positive tumors.

Taken together, these results show that MYB and MYBL1 are downregulated by p95HER2 in MCF-7 cells in a manner dependent on miR-503 and miR-221/222. Similar effects of these miRs on MYB family proteins are seen in other breast cancer cells.

### Knockdown of MYB family proteins upregulates TIMP2 expression

**Figure 6:**
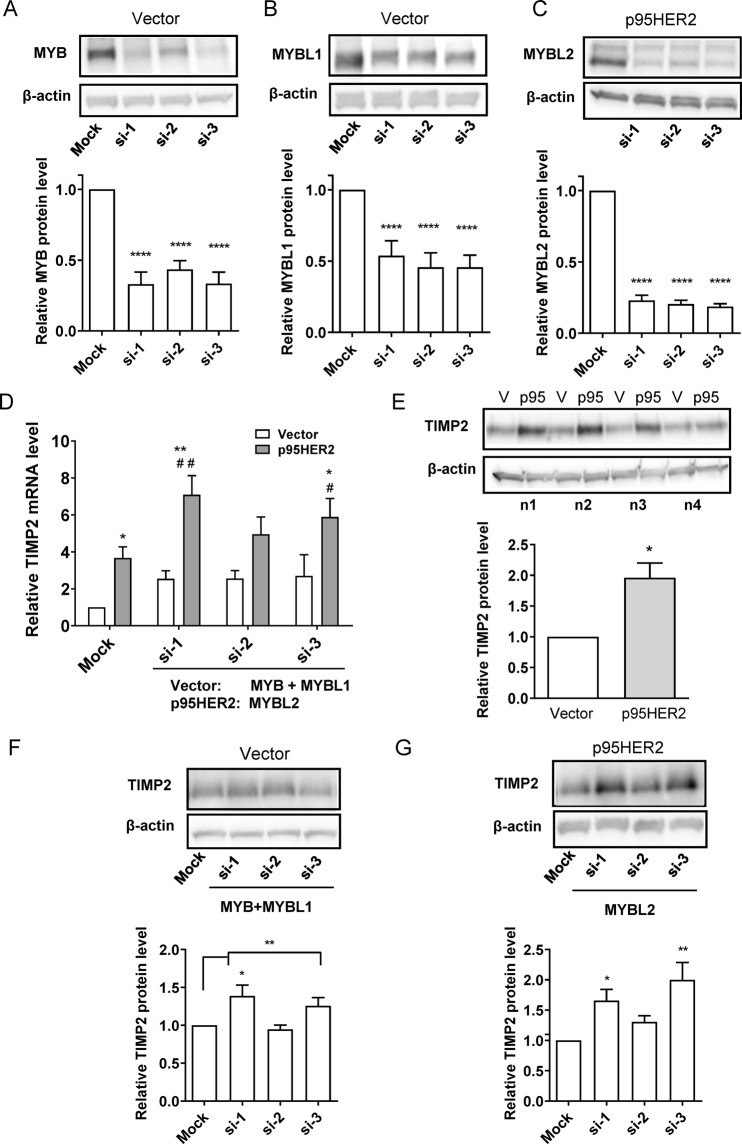
Knockdown of MYB family proteins and p95HER2 upregulation increase TIMP2 expression in MCF-7 cells. (**A–C**) Relative protein level of MYB (**A**), MYBL1 (**B**) and MYBL2 (**C**) after knockdown of MYB and MYBL1 (vector cells) or MYBL2 (p95HER2 cells). 48 h after induction and transfection with the indicated siRNAs, cells were lysed and subjected to Western blotting. Representative blots are shown, with β-actin as loading control. Graphs are mean values with S.E.M. error bars, based on 6–7 independent replicates per condition. M: mock siRNA, si, s2, s3: three different siRNAs for each MYB. ****p < 0.0001, One-way ANOVA with Dunnet post-test. (**D**) qPCR analysis of the TIMP2 mRNA level in vector- and p95HER2 cells after knockdown of MYB and MYBL1 (vector cells) or MYBL2 (p95HER2 cells). Reference genes are GAPDH and β-actin. Data are means with S.E.M. error bars, of 3 independent experiments. ***Significantly different from the corresponding mean values in vector cells, p < 0.05 and 0.01, respectively. ^###^Significantly different from the corresponding mean values in mock-transfected p95HER2 cells, p < 0.05 and 0.01, respectively. (**E**) Relative TIMP2 protein level is increased by p95HER2 expression in MCF-7 cells. The graph shows mean values with S.E.M. error bars, of 3 independent experiments. *Significantly different from the value in vector cells, p < 0.05. (**F,G**) Relative TIMP2 protein level is increased after knockdown of MYB and MYBL1 (vector cells) or MYBL2 (p95HER2 cells). ***Significantly different from the corresponding mean values in mock-transfected cells, p < 0.05 and 0.01, respectively.

MYB family transcription factors have very similar DNA binding motifs, yet regulate a partially distinct set of genes^18,40^. In order to identify downstream targets of the MYB proteins that would be significantly affected by p95HER2 expression, we compared previously published MYB family target genes^18^ to genes strongly regulated by p95HER2 expression^3^. This analysis revealed two proteins of interest, tissue inhibitor of matrix metalloproteases-2 (TIMP2) and Netrin-4 (NTN4), a laminin-related secreted molecule. While both TIMP2^41^ and NTN4^42^ have been linked to regulation of cancer invasiveness, and mRNA expression of both was increased both by p95HER2 and by MYB family protein knockdown (Fig. 6D and Suppl. Fig. 7, respectively), we focus here on TIMP2.

To analyze the effects on TIMP2 expression, we knocked down MYB and MYBL1 in empty vector expressing cells (simulating the effect of p95HER2 overexpression) and knocked MYBL2 down in p95HER2 cells. Robust knockdown of all three MYB family proteins was obtained using three different siRNA combinations (Fig. 6A–C). As noted above, the TIMP2 mRNA level was increased in p95HER2 cells compared to vector control cells and upon knockdown of MYB/MYBL1 or MYBL2 (Fig. 6D). Accordingly, the TIMP2 protein level was increased in p95HER2 cells compared to that in MCF-7 vector cells (Fig. 6E) and by MYB/MYBL1 knockdown in vector cells, and by MYBL2 knockdown in p95HER2 cells, albeit only by 2 of the 3 siRNA sets tested (Fig. 6F,G). Similar findings were obtained in T47D and SKBr-3 cells (Suppl. Fig. 8). Finally, consistent with the proposed pathway: miR-221/222/503 upregulation ->MYB and MYBL1 downregulation ->TIMP2 upregulation, overexpression of miR-221, -222, and -503 showed a tendency to increase TIMP2 expression (Suppl. Figs 4D and 5C).

Collectively, these results show that MYB family proteins negatively regulate the expression of TIMP2.

### Upregulation of miR-221/222 and knockdown of TIMP2 stimulates breast cancer cell motility

**Figure 7:**
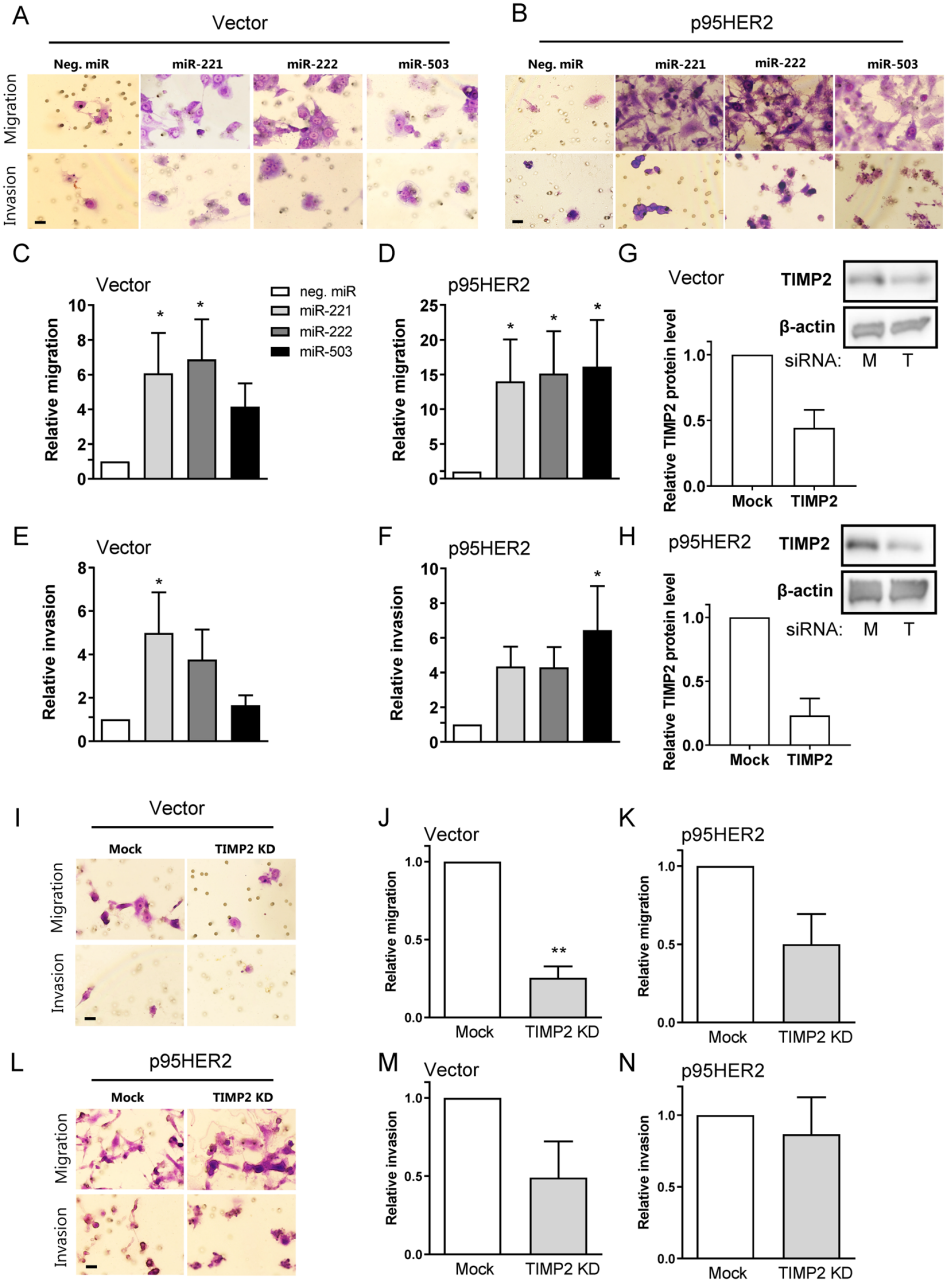
miR-221/222 and -503 overexpression increases, and TIMP2 knockdown decreases, cell motility. (**A,B**) Representative images of migrated (top panels) and invaded (bottom panels) cells for MCF-7 vector (**A**) and p95HER2 (**B**) cells. 48 h after induction and transfection with negative control miR (neg. miR), miR-221, miR-222, or miR-503, cells were resuspended in media containing 1% FBS and seeded for migration (non-coated) and invasion (coated) chambers placed in media containing 10% FBS for 24 h. Migrated/invaded cells were counted (20–80 images pr. membrane). Scale bar: 20 µM. (**C,D**) Quantification of migrated MCF-7 vector (**C**) and p95HER2-expressing cells (**D**). (**E,F**) Quantification of invaded MCF-7 vector- (**E**) and p95HER2 expressing cells (**F**). Data in (**C,F**) is shown as mean values relative to neg. miR +/− S.E.M., n = 3 for each cell line and condition. *Significantly different from neg. miR, p < 0.05, One-way ANOVA followed by Dunnet’s post-test. (**G,H**) Relative protein level of TIMP2 in vector (**G**) and p95HER2 (**H**) after knockdown of TIMP2. 48 h after induction and transfection with mock or TIMP2 siRNA, cells were lysed and subjected to Western blotting. Representative blots are shown, with β-actin as loading control. Graphs are mean values with S.E.M. error bars, n = 2–3 per condition. M: mock siRNA, T: TIMP2 siRNA. (**I–L**) Representative images of migrated (top panels) and invaded (bottom panels) cells for vector (**I**) and p95HER2 (**L**). 48 h after induction and transfection with mock or TIMP2 siRNA, cells were resuspended and seeded for migration/invasion as described in A + B. Scale bar: 20 µM. (**J,K**) Quantification of migrated MCF-7 vector (**J**) and p95HER2-expressing cells (**K**). (**N,M**) Quantification of invaded MCF-7 vector- (**N**) and p95HER2-expressing cells (**M**). Data in J-K and N-M is shown as mean values relative to mock +/− SEM, n = 3 (vector), 4 (p95HER2). **Significantly different from mock, p < 0.01, Student’s *t*-test.

The above results point to a signaling cascade in which p95HER2 upregulates the expression levels of miR-221, -222, and -503, resulting in reduced expression of their targets MYB and MYBL1, in turn increasing the expression of TIMP2. Given the proposed involvement of these miRs^14^ and TIMP2 in regulating cancer cell invasiveness^41^ we hypothesized that this axis would contribute to the invasiveness of p95HER2 expressing breast cancer cells^2,3^. We therefore tested the impact of miR mimics and TIMP2 knockdown on invasiveness of MCF-7 vector and p95HER2 overexpressing cells (Fig. 7). In Boyden chamber assays, miR-221, and miR-222 mimics potently increased migration in both vector- and p95HER2 expressing MCF-7 cells. The miR mimics also tended to increase invasion in both cell lines, although this only reached statistical significance for miR-221 in vector cells (Fig. 7A–F). miR-503 mimics increased both migration and invasion, in p95HER2 cells only. Finally, knockdown of TIMP2 (50–80% knockdown achieved, Fig. 7G,H) decreased migration and invasion in vector cells, and tended to decrease migration in p95HER2 cells (Fig. 7I–N). Again, a similar trend was seen in T47D and SKBr-3 cells (Suppl. Fig. 9).

Collectively, these results show that increased levels of miR-221, -222, and -503 increase MCF-7 cell migration and invasion. Conversely, knockdown of TIMP2 showed a strong tendency to decrease migration and invasion in MCF-7, T47D, and SKBr-3 cells, with the magnitude of this effect varying between breast cancer cell lines.

## Discussion

Despite the major societal burden of p95HER2-expressing breast cancers and the increased aggressiveness of these cancers compared to those expressing the full-length HER2 variant^3,4^, little is known regarding the functional differences between these two receptor variants. HER2 and p95HER2 induce similar downstream signaling pathways, leading to a qualitatively similar spectrum of changes in gene expression^3^. However, p95HER2 overexpressing cancer cells have a distinct protein secretome compared to that of overexpressing full-length HER2^33^, and p95HER2 expression correlates with lymph node metastasis^43^. In this work, we tested the hypothesis that p95HER2- and full-length HER2-only overexpressing cancers exhibit distinct patterns of miRNA expression, which may serve both as diagnostic tools and as starting points for gaining new mechanistic insight into these differences. Using small RNA sequencing, we provide evidence that HER2 and p95HER2 elicit distinct changes in miRNA profile in MCF-7 cells. Specifically, we identify a subset of miRNAs which are profoundly affected by p95HER2, with little effect of HER2. Notably, compared to HER2 expressing cells, the miRNA profile of p95HER2 cells was shifted toward that of basal subtype cancers, and overlapped highly with that previously identified for cancers with ERK1/2 hyperactivation^36^.

Two major upregulated miRNAs in p95HER2 cells were miR-221 and -222, which have been shown to contribute to breast cancer aggressiveness through multiple pathways^11–13^. Downregulation of ESR1 by miR-221/222 was previously reported^12^, as was downregulation of ESR1 by p95HER2^44^. Here, we show that p95HER2 downregulates ESR1 at least in part via miR-221 and -222. It is notable that upregulation of these miRNAs by full-length HER2 was marginal compared to that by p95HER2. We therefore suggest that the miR-221 and -222 upregulation in HER2-positive primary human breast cancer tissue^11^ is predominantly related to p95HER2 expression in a subset of these cancers. This is clinically important, as miR-221-HER2 co-expression was shown to be a negative prognostic marker for distant metastasis-free survival^14^ and as p95HER2 expression is associated with tamoxifen resistance^44^. It is also intriguing that in HER2 expressing cancers classified as ERα-negative, prediction of trastuzumab responsiveness was uncoupled from the HER2 mRNA level^45^. In light of the findings in the present work, this may suggest that the HER2+ /ESR1- patients are the subgroup with high p95HER2 levels.

A key finding of our work is the identification of a signaling axis of p95HER2 upregulation - miR-221/222 and miR-503 upregulation - MYB and MYBL1 downregulation - TIMP2 upregulation – increased cell motility. The miR-221/222/503 – MYB/MYBL1 - TIMP2 – cell motility axis was recapitulated in both T47D cells (Luminal A subtype) and SKBr-3 cells (HER2-positive subtype), suggesting a the signaling axis is universal rather than cell line/breast cancer subtype specific. Additionally, several elements of this axis have been shown or suggested previously in various cancers. Thus, miR-221 was suggested to negatively control MYBL1 in liver cancer, based on the miR-221-dependent downregulation of relative luciferase activity by the MYBL1 3′UTR^27^, and RNA-seq analysis in MCF-7 cells also indicated downregulation of MYB and MYBL1 by miR-221/222^46^. MYBL1 mRNA expression was previously shown to be downregulated upon p95HER2 expression^3^, Suppl. Table), and TIMP2 was shown to be upregulated by p95HER2^3^, Suppl. Table). Similar to upregulation of p95HER2 and miR-221/222 and -503, knockdown of endogenous MYB family proteins increased TIMP2 mRNA (MCF-7 and p95HER2 MCF-7 cells) and protein (MCF-7, p95HER2 MCF-7, T47D, and SKBr-3 breast cancer cells) expression. Curiously, a previous microarray analysis suggested that both a MYB repressor (consistent with our findings) and MYBL2 overexpression (counter to our findings) increased TIMP2 expression in MCF-7 cells^18^. This likely underscores that MYB proteins are highly context dependent in their impact on target gene regulation and effects of their exogenous overexpression can differ from that of endogenous MYB proteins^47^. In glioma cells, TIMP2 was negatively regulated by miR-221/222 and this was proposed to favor invasiveness^48^. Thus, the net effect of miR-221/222 on TIMP2 appears to be cancer type specific, and we speculate that this reflects the expression levels of other regulators such as the MYB family proteins. Similar to our findings, miR-503 was upregulated in esophageal^49^ and colorectal^50^ cancers, where it promoted tumor progression and correlated with aggressiveness/tumor size. On the other hand, miR-503 was downregulated in osteosarcoma cells. Interestingly however, this contributed to MYB *up*regulation, corroborating our finding that MYB is negatively regulated by miR-503^22^. Recently, a central role for the miR-426/503 cluster in mammary gland involution was demonstrated, via a mechanism involving miR-503-mediated targeting of Bcl-2^51^. As we previously showed that Bcl-2 is strongly downregulated in p95HER2 overexpressing cells^52^, this suggests that this effect may be dependent on miR-503 as well. Finally, MYB, MYBL1 and MYBL2 were reported to be upregulated by ESR1 signaling in breast cancer cells^20,34^. This would suggest that the MYB and MYBL1 downregulation in the present work could in part be secondary to reduced ESR1 signaling – although this may seem less likely given the lack of downregulation of MYBL2.

Our data show that upregulation of miR-221, -222, and -503 increases cell migration and invasion. It is interesting that both migration and invasion appear to be more potently increased by the miRs in p95HER2 overexpressing cells, which already have elevated levels of these miRs compared to vector controls. This suggests that the miRs synergize with other p95HER2-induced changes to strongly favor cell motility. Conversely, knockdown of TIMP2 tended to decrease both migration and invasion, in all four cell lines studied.

**Figure 8:**
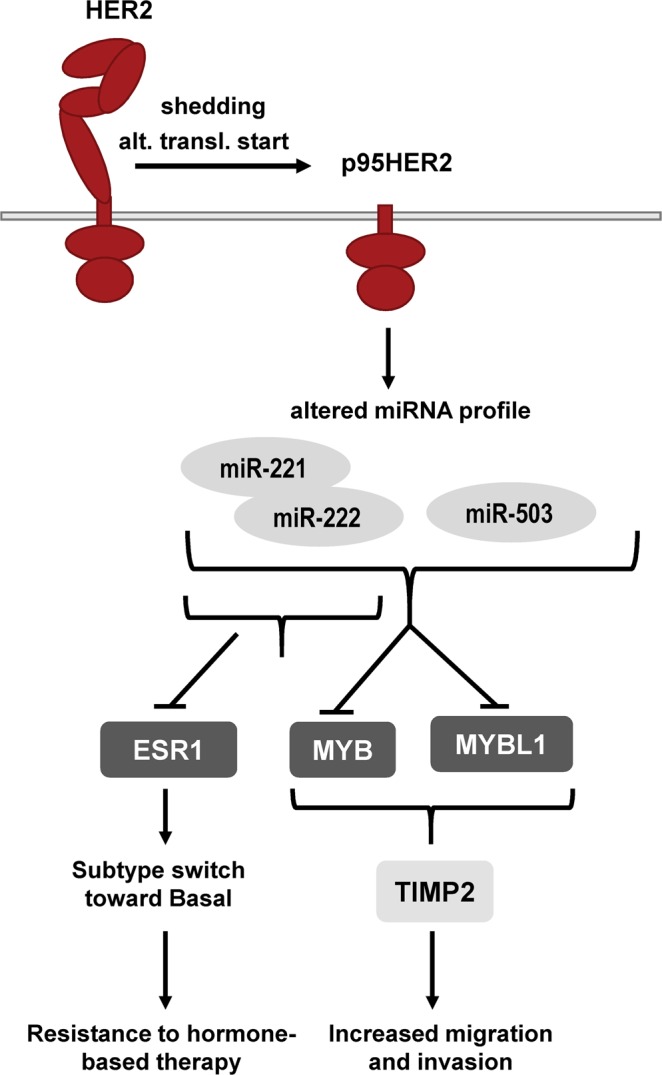
Proposed working model. The scheme illustrates the proposed sequence of events during a shift from HER2 only to p95HER2 expressing breast cancer. ‘‘alt. transl. start’’: alternative translation startsite, one of the pathways through which p95HER2 expression may be increased in breast cancer (the other being protease-activated shedding of the extracellular domain). See text for details.

A working hypothesis based on our findings is shown in Fig. 8: Elevated expression of p95HER2 relative to HER2 leads to a shift in miRNA profile involving increased levels of miR-221/222 and miR-503. miR-221/222 former potently downregulate ESR1, reflected in a shift toward the Basal breast cancer subtype. Both miR-221/222 and miR-503 contribute to downregulation of MYB and MYBL1 expression, in turn leading to increased TIMP2 expression, favoring increased invasion and metastasis.

In conclusion, our work reveals distinct differences between HER2 and p95HER2-induced miRNA expression profiles in breast cancer cells, with p95HER2 eliciting a shift toward the basal subtype. Additionally, we show that p95HER2 elicits ESR1 downregulation via miR-221/222, and we identify a novel, potentially therapeutically important pathway leading to MYB and MYBL1 downregulation by p95HER2 through miR221/222 and miR-503 upregulation. Downregulation of MYB family proteins in turn leads to TIMP2 upregulation, which likely contributes to the p95HER2 phenotype, at least in part via effects on cell migration and invasion.

## Materials and Methods

### Reagents

Antibodies against MYBL1 and β-actin were from Sigma-Aldrich (St. Louis, MO, USA). Total HER2 antibody was from Biogenex (CA, USA), antibodies against phospho-Tyr1221/1222 HER2 and TIMP2 were from Cell Signaling Technology (MA, USA). Antibodies against MYB and MYBL2 were from Merck Milipore (Darmstadt, Germany) and Santa Cruz Biotechnology (Dallas, Texas), respectively. Mouse monoclonal ESR1 antibody (6F11) was from Novocastra (Newcastle upon Tyne, England). Secondary antibodies for Western blotting (goat anti mouse and goat anti rabbit) were from DAKO (Denmark). miRNA mimics were from SwitchGear Genomics, Carlsbad, CA, USA: hsa-miR-222-3p - MIM0282, hsa-miR-503 - MIM0440, hsa-miR-769-5p - MIM0684, hsa-miR-361-3p - MIM0336, hsa-miR-221-3p- MIM0281, hsa-miR-149-5p - MIM0194, hsa-miR-22-3p - MIM0053, hsa-miR-27a-5p - MIM0070, hsa-miR-28-3p - MIM0074, hsa-miR-29a-3p - MIM0078, hsa-miR-92b-3p - MIM0110, hsa-miR-132-3p - MIM0157, hsa-miR-146b-5p - MIM0184, hsa-miR-296-3p - MIM0289, non-targeting control - MIM9002. MYB family siRNAs were from Sigma-Aldrich: MYB: siRNA1: CACCAUUCUGGAUAAUGUU[dT][dT]; siRNA2: GCGAAUAAAGGAAUUAGAA[dT][dT]; siRNA3: GAA AUGCCUUCUUUAACUU[dT][dT], MYBL1: siRNA1: CAAGCAAGAAGAUAUCUGA[dT][dT], siRNA2: GUAUCAGUAUGUGUCACCU[dT][dT], siRNA3: CCUACUAUUAGAAGAUCUA[dT][dT], negative siRNA control: SIC001, MYBL2: siRNA1: CAGAAACGAGCCUGCCUUA[dT][dT]; siRNA2: GCUUCUUGAGCGAGUCCAA[dT][dT], siRNA3: CUGUUAAGACCCUGCCCUU[dT][dT]. TIMP2 siRNA was an esiRNA mixture from Sigma-Aldrich (cat # EHU017331).

### Cell culture and transfection

All cell lines were maintained at 37 °C, 5% CO_2_. The MCF-7 cell lines inducibly expressing HER2 and p95HER2 were previously described^3,37,53^. For sequencing experiments, MCF7 Tet-Off cells were maintained in DMEM/F-12 (1:1) (Gibco, Denmark) supplemented with 10% fetal bovine serum (FBS; Sigma-Aldrich, St. Louis, MO, USA), 4 mM L-glutamine (Sigma-Aldrich), 0.2 mg/ml G418 (Gibco), 1 µg/ml doxycycline (Sigma-Aldrich) (hereafter denoted complete medium) at 37 °C and 5% CO_2._ Induction of HER2 and p95HER2 (611-CTF version of p95HER2)^3^ was performed by detaching cells with trypsin and washing with doxycycline-free medium. For transfection with miRNA mimics, cells were washed free of tetracycline and seeded in 96-well plates. 48 h later, transfection with 40 nM miRNA mimics was performed using DharmaFECT DUO (Dharmacon, Lafayette, CO, USA), and measurements were made after another 48 h. For siRNA mediated knockdown, cells were seeded in 6-well plates the day before transfection. On the day of transfection, cells were washed free of tetracycline, and transfection with 25 nM (MYB, MYBL1, MYBL2) or 10 nM (TIMP2) siRNA performed with Lipofectamine 2000 (Waltham, MA, USA). 48 h later, cells were processed for qPCR analysis or Western blotting as described below. T47D cells were maintained in RPMI-1640 (Sigma-Aldrich) supplemented with 10% FBS, 1% penicillin/streptomycin, and 5 µg/mL bovine Insulin-Transferrin-Selenium. SKBr-3 cells were maintained in DMEM (Gibco) containing 4.5 g/L glucose, 3.97 mM L-glutamine and 1 mM pyruvate, supplemented with 10% FBS and 1% penicillin/streptomycin. Both cell lines were transfected with miR mimics and siRNAs as described above.

### Small RNA sequencing and analysis

Cells were lysed in Trizol (Life Technologies) at 15, 36 or 60 h after HER2 or p95HER2 induction and for un-induced controls. Small RNA sequencing libraries were prepared for two biological replicates using the NEBNext Multiplex Small RNA Library Prep Set for Illumina (New England Biolabs, Ipswich, MA, USA). Samples were sequenced on a MiSeq with the MiSeq Reagent Kit v3 (Illumina, San Diego, CA, USA). Sequences were aligned against the hg19 human genome assembly using Novoalign with settings -a AGATCGGAAGAGCACACGTCT -l 16 -h -1 -1 -t 90 -g 50 -x 15 -o SAM -o FullNW -r All 51 -e 51 and miRNA expression was quantified using custom Perl scripts. Count data were filtered prior to differential expression analysis to remove lowly expressed miRNAs. The minimal required sum of counts was set to 50, with at least 1 sample having a count of more than 10. Filtered data were normalized using the edgeR package, and differentially expressed miRNAs were found using the glmFit function in edgeR, between the dox plus and dox minus matched samples. A miRNA was considered to be differentially expressed if the p-value was < 0.05 and the FDR < 0.1. For hierarchical clustering, mean-centered log_2_ (cpm + 2) was used as expression values.

### qPCR analysis

RNA extracted for RNA Sequencing was reverse transcribed and cDNA was diluted 1:10 before quantification with iTaq SYBR green supermix (BioRad) according to the manufacturers’ instructions in 15 µl reactions on a CFX96 instrument (BioRad). Primers (sequences in Suppl. Table 1) were validated with synthetic templates. qRT-PCR expression data were normalized to the selected reference gene (SNORD48).

For analysis of mRNA levels of NTN4 and TIMP2, vector- and p95HER2 MCF-7 cells were transfected with siRNA against MYB, MYBL1, and MYBL2 as described above, and after 48 h, mRNA was extracted using the NucleoSpin RNA II kit (Macherey-Nagel, Düren, Germany). mRNA was reverse transcribed using Superscript RTII and random primers (Invitrogen, Carlsbad, CA, USA). SyBR green qPCR was performed in triplicates on each cDNA sample using 1 µg cDNA and 19 µl qPCR master mix (Stratagene), 2 µM forward and reverse primers, and 30 nM reference dye (ROX). Primer sequences are in Suppl. Table 1. qPCR analysis was performed in a Stratagene MX4000 Real Time Thermal cycler (95 °C 10 min; 40 × [95 °C 30 s, 55 °C 1 min, 72 °C 30 s]). Melting curves always confirmed the presence of only one amplicon. Data were analyzed using the Pfaffl method, with GAPDH and β-actin as reference genes.

### Bioinformatic analyses

Correlations between miRNA and mRNA levels in HER2 and p95HER2 expressing cells were analyzed using data from the present study and from *GSE68256*^3^, respectively, while breast cancer patient data were analyzed using the TCGA cohort from^35^. We identified 18 up-regulated and 10 down-regulated miRNAs, which were significantly deregulated in p95 samples but not in HER2 samples. We next calculated a miRNA score = *mean(up-regulated miRNAs) − mean(down-regulated miRNAs)* for all samples in the TCGA cohort^35^, revealing a significantly (Wilcoxon) greater score for samples from basal compared to luminal tumor types (PAM50). Similar calculations were performed for mRNA datasets. The raw excel files from geo accession GSE68256^3^ were downloaded and normalized using the gcrma package in R. A custom annotation file was downloaded from http://brainarray.mbni.med.umich.edu/brainarray/default.asp and used to map the probes to entrezgene identifiers. The limma package was used to find differentially expressed genes between the plus dox and minus dox samples. A gene was considered differentially expressed if the B-H adjusted p-value was less than 0.001 and the absolute log2 fold change >0.8. In order to analyse *GSE68256* we filtered the list of significantly regulated mRNAs in the p95 samples to mRNAs regulated more than 2 Log fold changes to the regression line (blue line, Fig. 2C). There were 50 downregulated and 53 upregulated mRNAs in the mRNA score, calculated as = *mean(filtered up-regulated mRNAs)*− *mean(filtered down-regulated mRNAs)*. As for the miRNA scores, the mRNA scores were higher in the basal samples.

### SDS-PAGE and Western blotting

Cells were lysed in SDS lysis buffer (0.1 M TRIS pH 7.5, 10% SDS, 1 mM NaVO_3_, and Complete protease inhibitor mix (Roche)) heated to 90 °C. Protein concentrations were determined using DC assay (BioRad, Hercules, CA). Samples were mixed with sample loading buffer (Life Technologies). Samples and BenchMark ladder (Life Technologies) were separated in BioRad Tris/Glycine/SDS running buffer on pre-made 10% Bis-Tris gels (BioRad). Proteins were transferred to PVDF membranes using Trans-Blot Turbo transfer system (BioRad), Ponceau S stained, and blocked for 1 h at 37 °C in 5% non-fat dry milk in TBST (0.01 M Tris/HCl, 0.15 M NaCl, 0.1% Tween 20, pH 7.4). Densitometric analyses were carried out using UN-SCAN-IT 6.1 software (Silk Scientific, Orem, Utah).

### Luciferase reporter assay

Cells were washed and seeded in 96-well plates 48 h before transfection. Transfections were performed using 40 ng of different constructs of MYBL1 3′UTR containing psiCHECK2 (Promega, Madison, WI, USA) vector DNA, 40 nM miRNA mimic and Dharmafect Duo. 48 h after transfection, cells were lysed and luciferase activities measured using Dual-Luciferase Reporter Assay System (Promega). psiCHECK2 containing 3′UTR of MYBL1 under control of Renilla luciferase was a kind gift from Pascal Pineau, Institut Pasteur, France. Site directed mutagenesis was performed using QuikChange Lightning Site-Directed Mutagenesis Kit (Agilent Technologies, Santa Clara, CA, USA), with the primers mut1: 5-GCCAGACATAACATGTGCGAGCCATACTTGCATGG-3, mut2: 5-GAACCAAGAGGCAGGAGACATATAGATATATACATATG-3 and mut3: 5-CACTTAGTGAAACCAATGATCGCCGCAAACTCATACTGGATC-3.

### Cell migration and invasion analyses

Cell migration and invasion assays were executed using 8.0 µM Control and Matrigel-coated inserts (Corning, MA, USA). Cells were transfected (siRNA or miR mimics) for 48 h and 15.000–105.000 cells were seeded in media containing 1% serum and incubated 20–24 h at 37 °C/5% CO_2_ with a 10% serum chemo attractant. Non-migrated/invaded cells were removed using a cotton swab and migrated/invaded cells were fixed in methanol for 60 min. Fixed cells were stained with 30% Giemsa (Sigma-Aldrich) in Gurr buffer (Life Technologies) for 30 min. Cells were counted manually from 20–80 images pr. membrane, using an Olympus microscope with 40X magnification.

### Statistical analysis

For sequencing and bioinformatics data see above. Statistical analysis of all other data was carried out using SPSS software. One-way ANOVA followed by Dunnett (at equal variance) or Dunnett C (at unequal variance) post-tests were employed, and p < 0.05 was taken to indicate a statistically significant difference.

## Supplementary information

Legends for supplementary tables and figures

Supplementary table 1

Supplementary table 2

## Data Availability

The datasets used in the current study are available from the corresponding author on reasonable request.
