# Supplementary material for: HER2 and p95HER2 differentially regulate miRNA expression in MCF-7 breast cancer cells and downregulate MYB proteins through miR-221/222 and miR-503
Source: Sci Rep. 2019 Mar 4;9:3352. doi: 10.1038/s41598-019-39733-x (PMC6399295; doi:10.1038/s41598-019-39733-x)

Supplementary table- and figure legends for:

Supplementary figures for:

HER2 and p95HER2 differentially regulate miRNA expression in MCF-7 breast cancer cells and downregulate MYB proteins through miR-221/222 and miR-503

Andrej Gorbatenko[1,2], Rolf Søkilde[3], Ester E. Sorensen[2], Inga Newie[3], Helena Persson[3], Beatriz Morancho[4], Joaquin Arribas[4,5,6], Thomas Litman[7], Carlos Rovira[3], and Stine Falsig Pedersen[2]*

[1]Department of Pathology, Icahn School of Medicine at Mount Sinai, New York, New York 10029, USA;[2]Section for Cell and Developmental Biology, Department of Biology, Faculty of Science, University of Copenhagen, Universitetsparken 13, DK-2100 Copenhagen, Denmark [3]BioCare, Strategic Cancer Research Program, Lund, Sweden; Department of Clinical Sciences Lund, Oncology and Pathology, Faculty of Medicine, Lund University, Lund, Sweden. [4]Preclinical Research Program, Vall d'Hebron Institute of Oncology and CIBERONC, 08035 Barcelona, Spain; [5]Department of Biochemistry and Molecular Biology, Universitat Autonoma de Barcelona, Campus de la UAB, Bellaterra, Spain (JA); [6]Institució Catalana de Recerca i Estudis Avançats, Barcelona, Spain (JA); [7]Department of International Health, Immunology and Microbiology, University of Copenhagen, Blegdamsvej 3B, DK-2200 Copenhagen, Denmark.

Supplementary tables

Suppl. Table 1: Primers used for confirmation of miRNA sequencing results

Suppl. Table 2: miRNAs significantly affected by p95HER2 but not by HER2

The table shows the LogFC values and p-values for the 28 miRNAs, expression of which was significantly altered by p95HER2 by not by HER2. Data are means of 2 independent biological replicates.

Supplementary figures

Suppl. Fig. 1. Induction of p95HER2 and HER2 in MCF-7 cells

Cells were washed free of doxycycline at time zero, and lysed for Western blotting at the times indicated. p-p95 and p-HER2 indicate blotting with anti-phospho-Tyr1221/1222 HER2 antibody, to indicate the level of activity of the respective receptors. β-actin is shown as a loading control. The blots represent 2 biological replicates.

Suppl. Fig. 2. Expression profiles of selected miRNAs in p95HER2 and HER2 expressing cells

The figures show expression profiles obtained by sequencing, compared to the corresponding expression patterns obtained by qPCR analysis, at time 15, 36 and 60 h after p95HER2/HER2 induction, of 11 miRNAs that were strongly up- or down regulated by p95HER2 signaling. Expression profiles for miR-503 and miR-221 and -222 are shown in Fig. 1C. Sequencing data are shown as log2 transformed relative read counts, normalized to counts in control samples, and are means from 2 biological replicates. qPCR data are shown as $\Delta\Delta$Cq values, and are from 2 biological replicates. Grey bars: p95HER2, white bars: HER2.

Suppl. Fig. 3. hMAPK miRNA profile

The hMAPK–miRNA signature from Miller et al. [1] was analyzed. 26 miRNAs were found in the overlap between the signature and the miRNAs passing expression filters in our dataset. Of these, 11 were in the signature down-regulated group (dark grey) and 15 in the signature up-regulated group (light grey). Seven of the miRNAs in the Miller hMAPK-miRNA signature were significantly affected in our p95HER2 60 h samples, 1 down-regulated (miR-149-5p) and six up-regulated (miR-221-3p, miR-222-3p, miR-146b-5p, miR-22-3p, miR23a-3p, miR-24-3p).

Suppl. Fig. 4. miR-221/222 mimics reduce expression of MYB and MYBL1 and tend to increase expression of MYBL2 and TIMP2 in T47D cells

T47D cells were transfected with 40 nM miR-221 and/or miR-222, miR-503, or negative control miRNA as shown, lysed, and subjected to Western blotting for MYB (A), MYBL1 (B), MYBL2 (C), and TIMP2 (D). Representative blots are shown for each protein and condition. Corresponding graphs show the relative protein level, as means with S.E.M. error bars, normalized to the β-actin loading control, and represent 3-5 independent experiments per condition. *,**: $p < 0.05$, 0.01 relative to neg-miR, one-way ANOVA with Dunnet post-test.

Suppl. Fig. 5. miR-221/222 mimics reduce expression of ESR1, MYB, and MYBL1 in T47D cells

T47D cells were transfected with 40 nM miR-221 and/or miR-222, miR-503, or negative control miRNA as shown, lysed, and subjected to Western blotting for ESR1 (A), MYB (B), MYBL1 (C), and MYBL2 (D). Data are summarized as means with S.E.M. error bars, normalized to the β-actin loading control, and represent 2 (A) and 4 (B-D) biological replicates. *: $p < 0.05$, repeated measures one-way ANOVA with Dunnet post-test.

Suppl. Fig. 6. PAM50 data analyzed for breast cancer subtype expression of HER2 and MYB proteins

The mean-centered expression values for HER2 (A), MYB (B), MYBL1 (C) and MYBL2 (D) from the TCGA PAM50 dataset were plotted for the different breast cancer subtypes. Number of samples was: Basal-like: 103, HER2-enriched: 58, Luminal A: 241, Luminal B, 137, Normal-like: 8. *P*-values are shown on the graphs for p.adjust < 0.001 (Holm adjustment, [2]).

Suppl. Fig. 7. The mRNA level of NTN4 in vector- and p95HER2 expressing cells is increased by MYB protein knockdown

qPCR analysis of the NTN4 mRNA level in vector- and p95HER2 cells after knockdown of MYB and MYBL1 (vector cells) or MYBL2 (p95HER2 cells). Reference genes are GAPDH and β-actin. Data are means with S.E.M. error bars, of 3 independent experiments. *: Significantly different from the corresponding mean values in vector cells, $p < 0.05$, one-way ANOVA with Dunnet post-test.

Suppl. Fig. 8. Knockdown of MYB family proteins increases TIMP2 protein expression in T47D and SKBr-3 cells

T47D cells are used as a model for Luminal A breast cancer cells and SKBr-3 cells as a model of HER2 overexpressing breast cancer cells. A-C. Relative protein level of MYB (A), MYBL1 (B) and TIMP2 (C) after knockdown of MYB and MYBL1 in T47D cells. D-E. Relative protein level of MYBL2 (D) and TIMP2 (E) after knockdown of MYBL2 in SKBr-3 cells. 48 h after induction and transfection with the indicated siRNAs, cells were lysed and subjected to Western blotting. Representative blots are shown, with β-actin as loading control. Graphs are mean values with S.E.M. error bars, based on 6-7 independent replicates per condition. M: mock siRNA, si, s2, s3: three different siRNAs for each MYB. ****: $p < 0.0001$, one-way ANOVA with Dunnet post-test.

Suppl. Fig. 9. TIMP2 knockdown decreases cell motility in T47D and SKBr-3 cells

A-B. Relative protein level of TIMP2 in T47D (A) and SKBr-3 (B) after knockdown of TIMP2. 48 h after transfection with mock or TIMP2 siRNA, cells were lysed and subjected to Western blotting. Representative blots are shown, with β-actin as loading control. Data represent 3 experiments in T47D and 2 in SKBr-3 cells. Graphs are mean values with S.E.M. error bars, *) significantly different from mock, p<0.01, Student's *t*-test. C+D. Representative images of migrated (top panels) and invaded (bottom panels) cells for T47D (C) and SKBr-3 (D). 48 h after transfection with mock or TIMP2 siRNA, cells were resuspended in media containing 1% FBS and seeded for migration (non-coated) and invasion (coated) chambers placed in media containing 10% FBS for 24 h. Migrated/invaded cells were counted (20-80 images pr. membrane). Scale bar: 20 µM. E+F. Quantification of migrated T47D (E) and SKBr-3 (F). G+H. Quantification of invaded T47D (G) and SKBr-3 (H). Data in E-H is shown as mean values relative to mock +/- SEM, n=X. *) significantly different from mock, p<0.05, Student's t-test.

References

1. Miller,P.C., Clarke,J., Koru-Sengul,T., Brinkman,J., & El-Ashry,D. A novel MAPK-microRNA signature is predictive of hormone-therapy resistance and poor outcome in ER-positive breast cancer. *Clin. Cancer Res.* **21**, 373-385 (2015).

2. Holm,S. A simple sequentially rejective multiple test procedure. *Scandinavian Journal of Statistics* **6**, 65-70 (1979).

Suppl. Figure 1

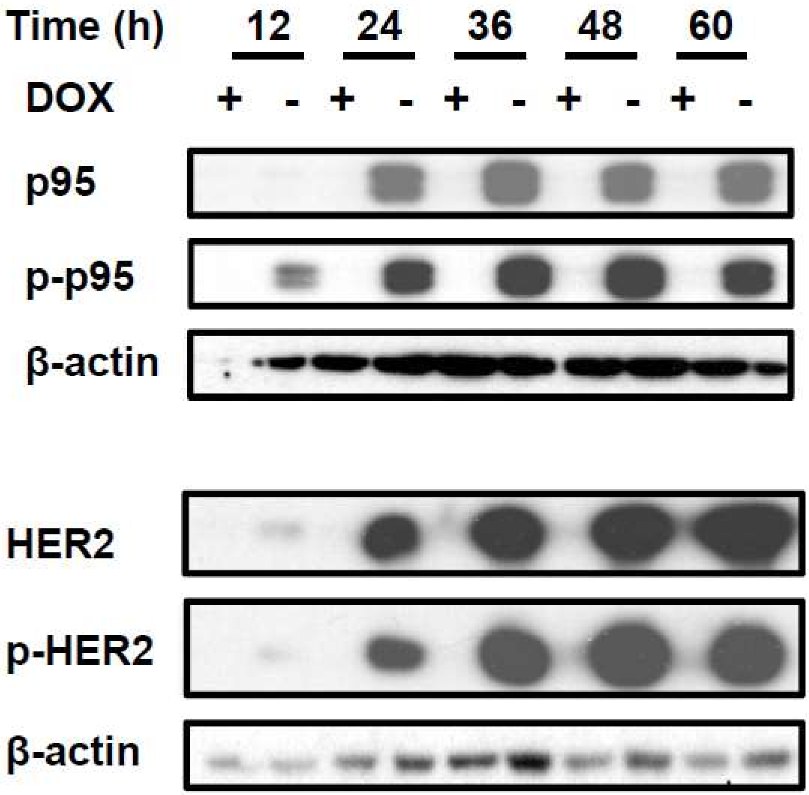

Suppl. Figure 2

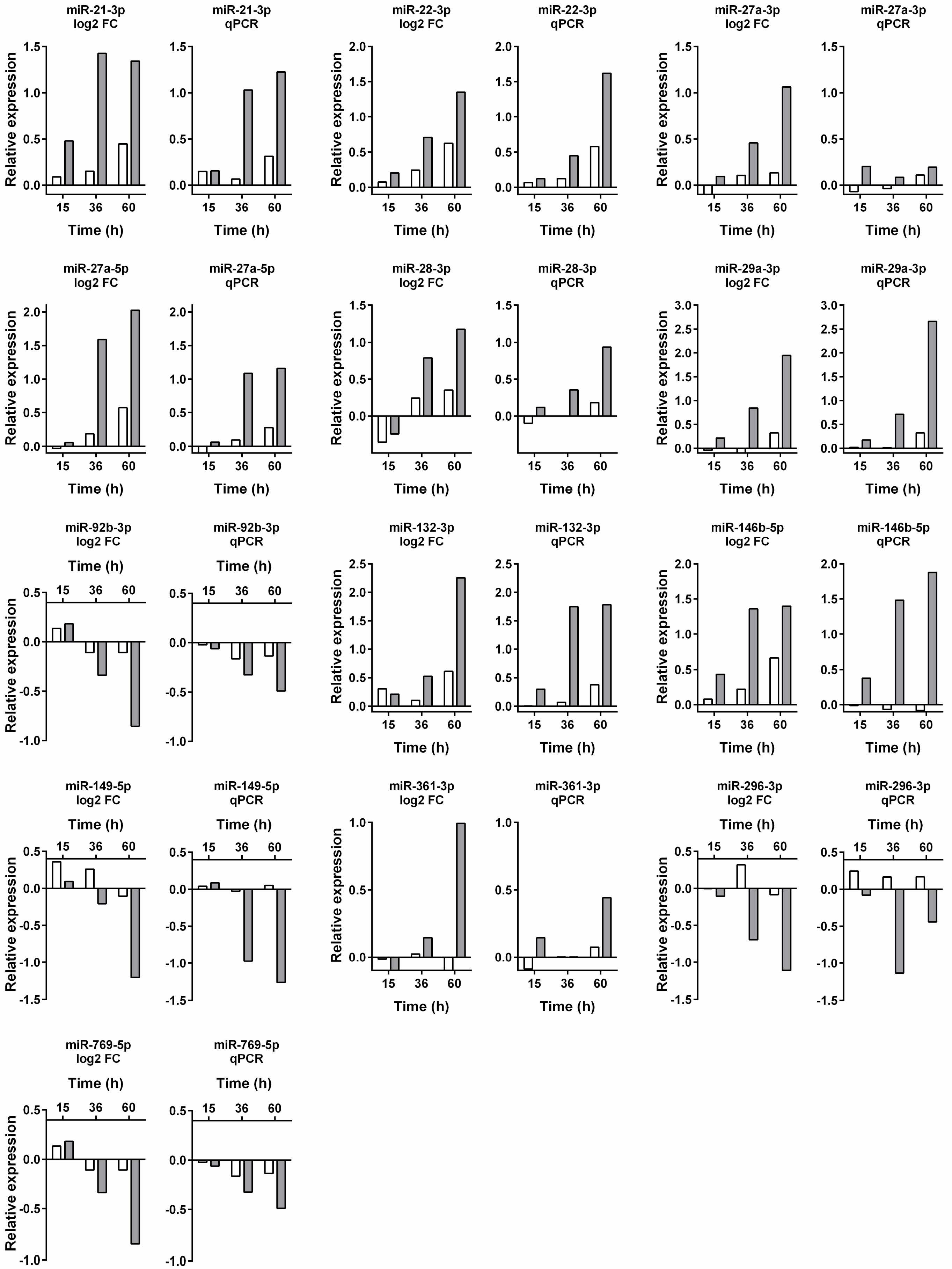

Suppl. Fig. 3.

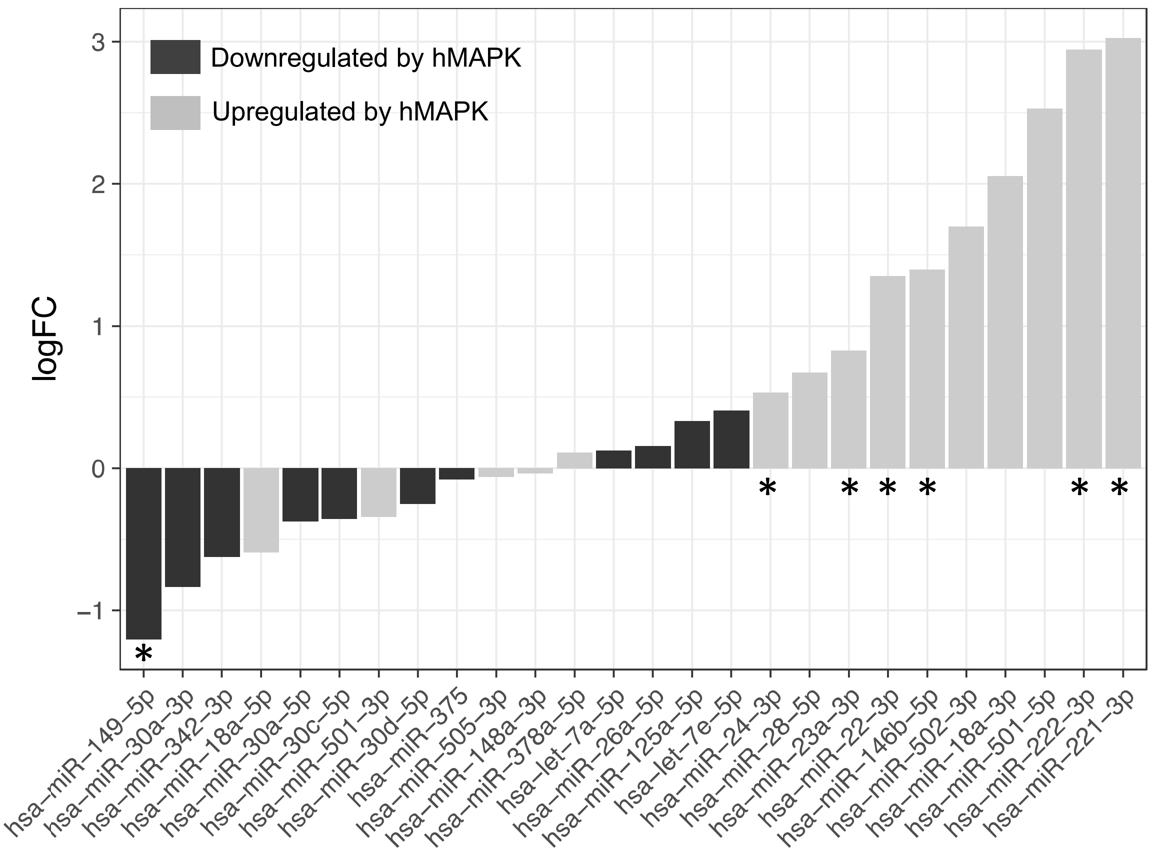

*): miRNAs significantly affected in the p95HER2 60 h group in the present dataset

Suppl. Fig. 4

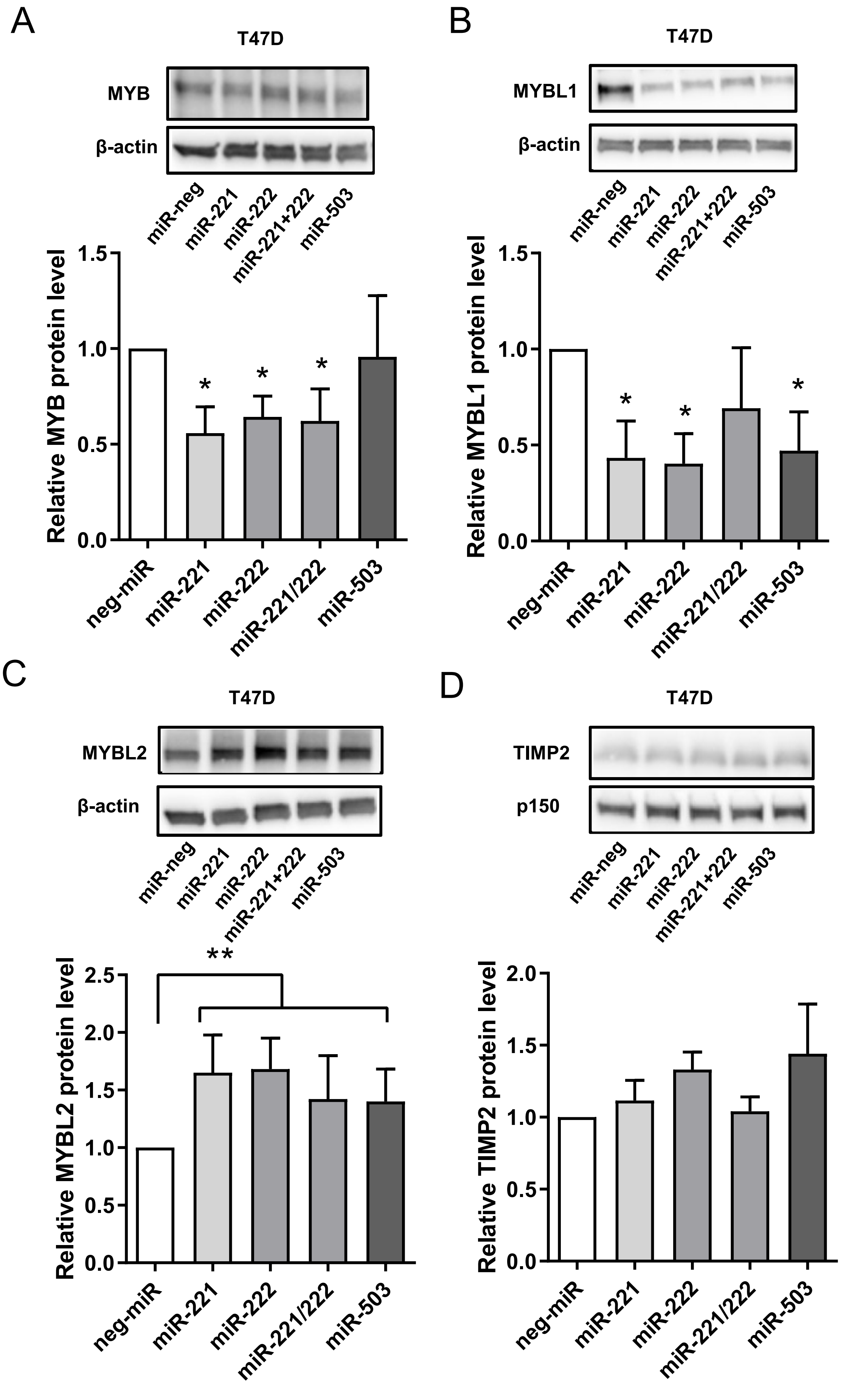
A
T47D
MYB
β-actin
miR-neg
miR-221
miR-222
miR-221+222
miR-503
Relative MYB protein level
1.5
1.0
0.5
0.0
neg-miR
miR-221
miR-222
miR-221/222
miR-503
B
T47D
MYBL1
β-actin
miR-neg
miR-221
miR-222
miR-221+222
miR-503
Relative MYBL1 protein level
1.5
1.0
0.5
0.0
neg-miR
miR-221
miR-222
miR-221/222
miR-503
C
T47D
MYBL2
β-actin
miR-neg
miR-221
miR-222
miR-221+222
miR-503
**
Relative MYBL2 protein level
2.5
2.0
1.5
1.0
0.5
0.0
neg-miR
miR-221
miR-222
miR-221/222
miR-503
D
T47D
TIMP2
p150
miR-neg
miR-221
miR-222
miR-221+222
miR-503
Relative TIMP2 protein level
2.0
1.5
1.0
0.5
0.0
neg-miR
miR-221
miR-222
miR-221/222
miR-503

Suppl. Fig. 5

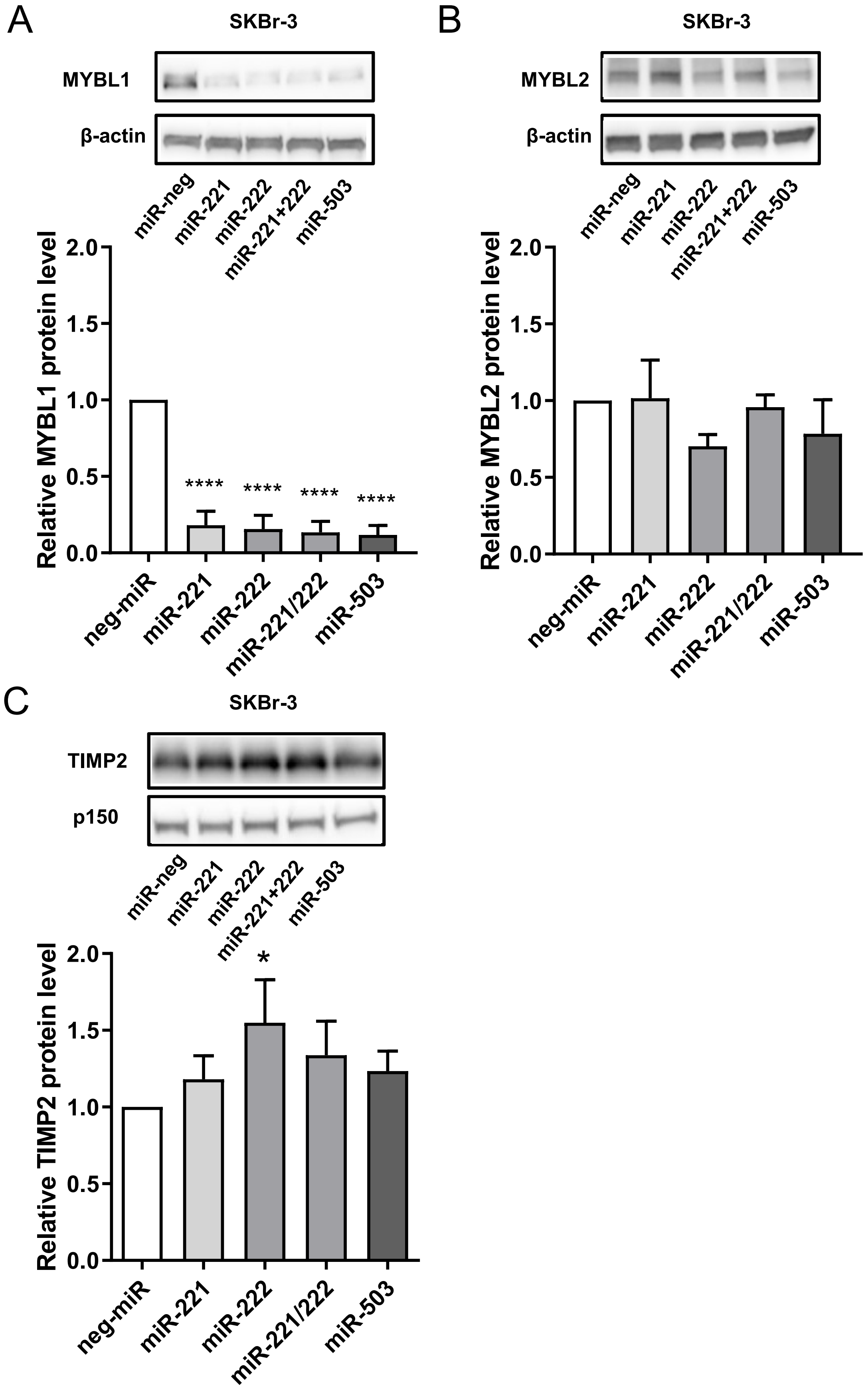
A
SKBr-3
MYBL1
β-actin
miR-neg
miR-221
miR-222
miR-221+222
miR-503
Relative MYBL1 protein level
2.0
1.5
1.0
0.5
0.0
****
****
****
****
neg-miR
miR-221
miR-222
miR-221/222
miR-503
B
SKBr-3
MYBL2
β-actin
miR-neg
miR-221
miR-222
miR-221+222
miR-503
Relative MYBL2 protein level
2.0
1.5
1.0
0.5
0.0
neg-miR
miR-221
miR-222
miR-221/222
miR-503
C
SKBr-3
TIMP2
p150
miR-neg
miR-221
miR-222
miR-221+222
miR-503
Relative TIMP2 protein level
2.0
1.5
1.0
0.5
0.0
*
neg-miR
miR-221
miR-222
miR-221/222
miR-503

Suppl. Figure 6

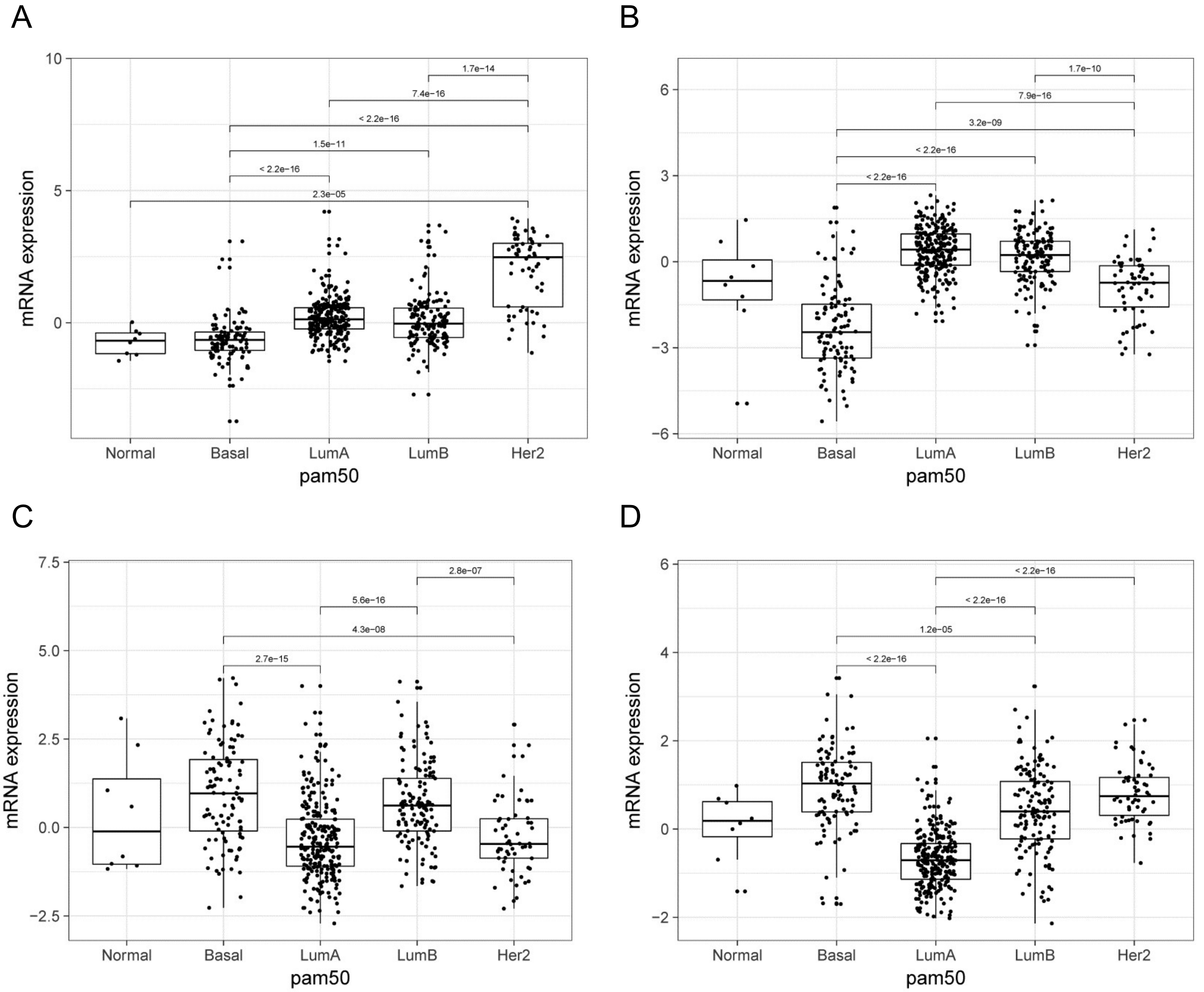

Suppl. Figure 7

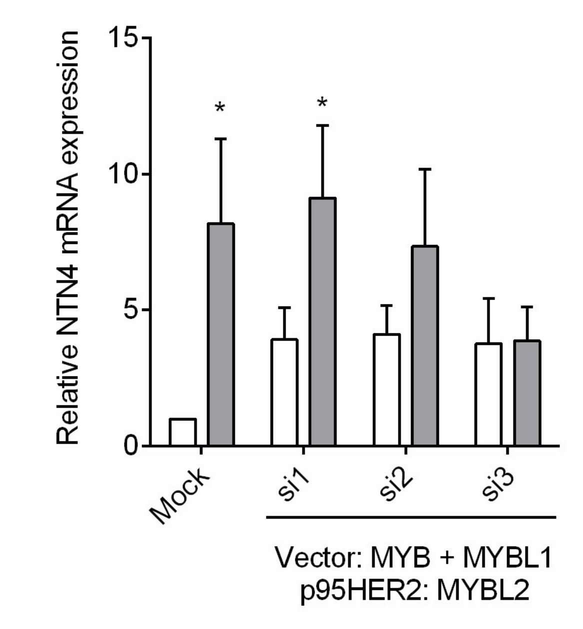

Suppl. Figure 8

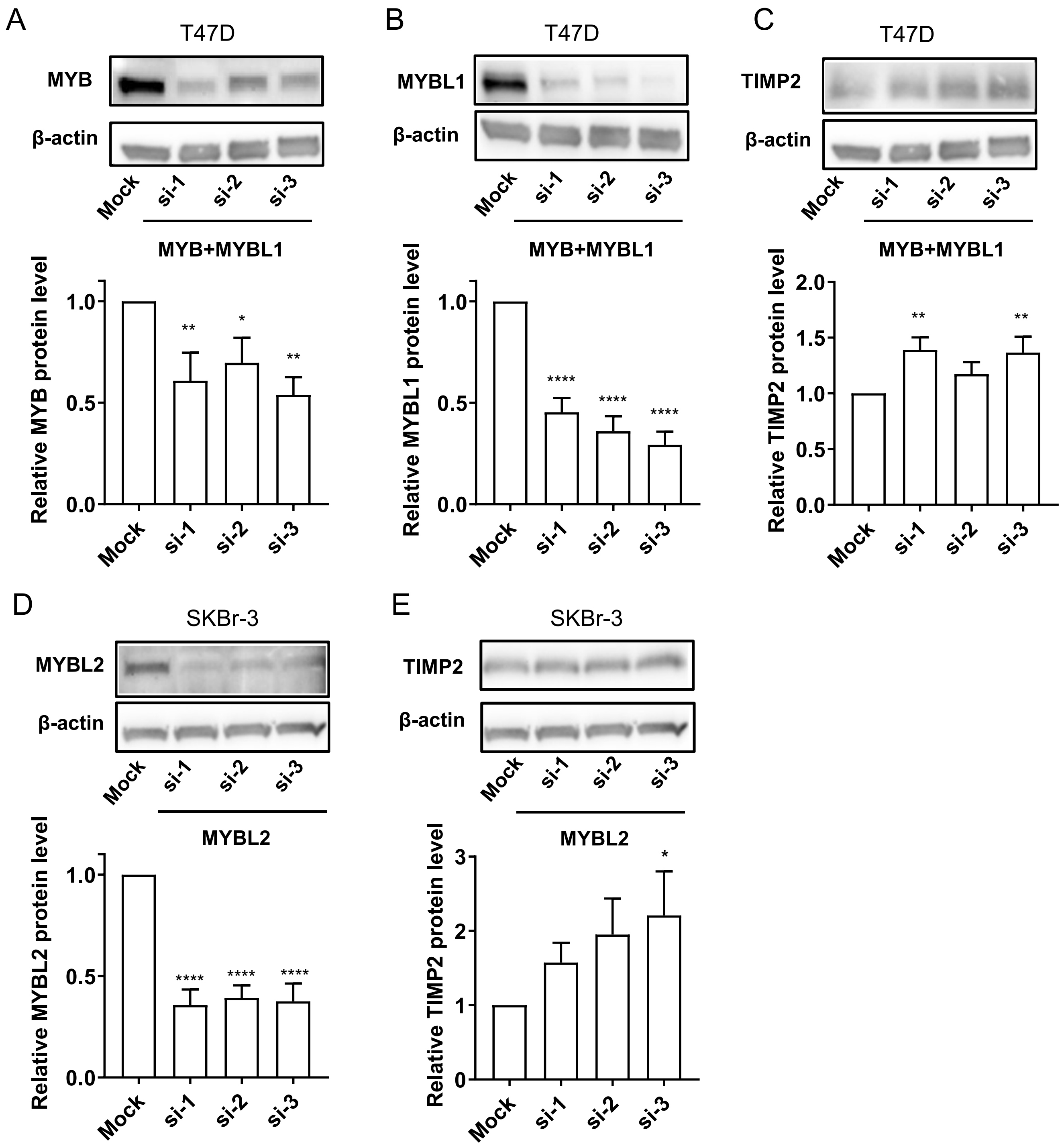

Suppl. Figure 9

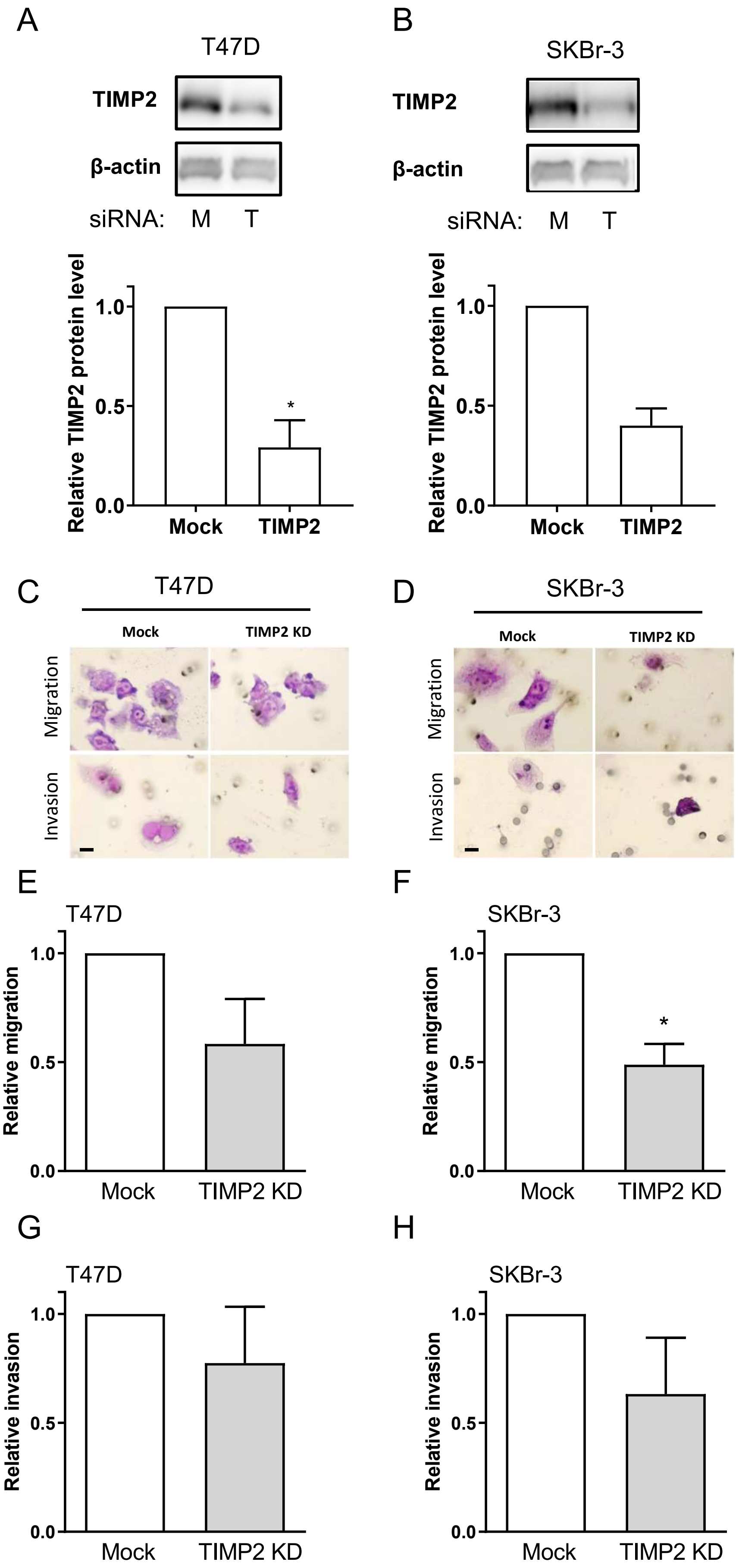

Supplement: Supplementary file 1 — Legends for supplementary tables and figures [file 41598_2019_39733_MOESM1_ESM.pdf]
